# Proteomic profiling of Arabidopsis nuclei reveals distinct protein accumulation kinetics upon heat stress

**DOI:** 10.1038/s41598-024-65558-4

**Published:** 2024-08-14

**Authors:** E. Muñoz-Díaz, I. Fuenzalida-Valdivia, T. Darrière, A. de Bures, F. Blanco-Herrera, M. Rompais, C. Carapito, J. Sáez-Vásquez

**Affiliations:** 1 Laboratoire Génome et Développement des Plantes (LGDP), UMR 5096, CNRS, 66860 Perpignan, France; 2 LGDP, UMR 5096, Univ. Perpignan Via Domitia, 66860 Perpignan, France; 3 Facultad de Ciencias de la Vida, Centro de Biotecnología Vegetal, Universidad Andrés Bello, 8370146 Santiago, RM Chile; 4 ANID - Millennium Institute for Integrative Biology (IBio), Santiago, Chile; 5 ANID - Millennium Science Initiative Program, Millennium Nucleus for the Development of Super Adaptable Plants (MN-SAP), 8331150 Santiago, Chile; 6 Laboratoire de Spectrométrie de Masse BioOrganique, IPHC UMR 7178, CNRS, Université de Strasbourg, Infrastructure Nationale de Protéomique ProFI - FR2048, Strasbourg, France

**Keywords:** nanoLC‒MS/MS, Heat stress, Nucleus, Nucleolus, Differential proteomics, Biochemistry, Plant sciences

## Abstract

Heat stress (HS) impacts the nuclear proteome and, subsequently, protein activities in different nuclear compartments. In *Arabidopsis thaliana*, a short exposure to 37 °C leads to loss of the standard tripartite architecture of the nucleolus, the most prominent nuclear substructure, and, consequently, affects the assembly of ribosomes. Here, we report a quantitative label-free LC‒MS/MS (Liquid Chromatography coupled to tandem Mass Spectrometry) analysis to determine the nuclear proteome of *A*rabidopsis at 22 °C, HS (37 °C for 4 and 24 h), and a recovery phase. This analysis identified ten distinct groups of proteins based on relative abundance changes in the nucleus before, during and after HS: Early, Late, Transient, Early Persistent, Late Persistent, Recovery, Early-Like, Late-Like, Transient-Like and Continuous Groups (EG, LG, TG, EPG, LPG, RG, ELG, LLG, TLG and CG, respectively). Interestingly, the RNA polymerase I subunit NRPA3 and other main nucleolar proteins, including NUCLEOLIN 1 and FIBRILLARIN 1 and 2, were detected in RG and CG, suggesting that plants require increased nucleolar activity and likely ribosome assembly to restore protein synthesis after HS.

## Introduction

As sessile organisms, plants continuously face diverse biotic and abiotic stresses. Global warming and the greenhouse effect are leading to an increase in the average temperature worldwide. An increase of 10°–15 °C above the optimum temperature is considered to be heat stress (HS). HS negatively affects plant growth and development, limiting the photosynthetic rate and germination efficiency^1^. This HS condition threatens the yield and production of crop species worldwide. However, plants have developed a series of response mechanisms defining the heat stress response (HSR)^2–4^.

One of the key events in HSR is the accumulation of heat shock proteins (HSPs). HSPs act as molecular chaperones, preventing unfolding of proteins, particularly during HS. They are classified according to their molecular weight as HSP100, HSP90, HSP70, HSP60 and small HSPs^5,6^. Transcription of HSPs is controlled by heat shock factors (HSFs)^5^. The most studied HSFs in *Arabidopsis thaliana* are class A HSFs, including HSFA1a or HSFA2, which are critical players in the response to HS. For instance, Arabidopsis HSFA2 shuttles from the cytoplasm to accumulate in the nucleus in response to HS^7^. In contrast, class B and C HSFs are less well characterized^8,9^. Similarly, the transcription factors bZIP18 and bZIP52, as well as heat shock factor-binding protein (HSBP), translocate from the cytoplasm to the nucleus in response to heat stress in plants^10^.

In addition to proteins, noncoding RNAs (ncRNAs) are involved in the response to HS^4^. In Arabidopsis, HS rapidly induces accumulation of the microRNA miR398, as well as increasing HSFs and HSP levels, whereas miR156 operates in recovery from HS^11,12^. Moreover, HTI1 and HTI2, two small-interfering RNAs (siRNAs), are involved in thermotolerance in Arabidopsis^13^. In addition, certain epigenetic marks seem to be involved in the response and adaptation to HS. For instance, HS increases global methylation in the Arabidopsis genome^14^. *Arabidopsis thaliana* mutants, for *DICER-LIKE 3* (*DCL3*) or *ARGONAUTE 4* (*AGO4*) genes, among others, involved in RNA-directed DNA methylation show increased sensitivity to HS^15^. Histone modifications are another factor involved in the response to HS, as some of these modifications are affected by HS in plants^16,17^. Apart from the changes in the transcriptome, proteome and epigenetic marks mentioned above, several effects have been described with regard to the shape, number and composition of different nuclear bodies in plants^18^. Specifically, the effect of HS on the nucleolus in plants has been explored^19,20^.

The nucleolus is the most prominent nuclear body. Its structure and function are well-characterised^21,22^. We previously showed that Arabidopsis nucleoli rapidly disorganize and disassemble in response to HS. Nevertheless, this disruption is reversible, as the structure of Arabidopsis nucleoli slowly recovers when the plants are returned to standard growth conditions^19^. In this context, disruption and reassembly of the nucleolus might affect the nuclear proteome. Here, we present a quantitative analysis of the nuclear proteome of Arabidopsis plants during heat treatment (37 °C) and during recovery conditions (22 °C after heat treatment). We describe ten groups of proteins according to their nuclear abundance changes during and after HS. This analysis identified significant accumulation of major nucleolar proteins, such as FIBRILLARIN 2 (FIB2), NUCLEOLIN 1 (NUC1) and a subunit of RNA polymerase I (RNA Pol I), in the recovery phase after HS. This striking phenomenon suggests the requirement of the nucleolar machinery to resume rRNA transcription and ribosome biogenesis after HS.

## Methods

### Plant materials and growth conditions

All lines were derived from the *Arabidopsis thaliana* Columbia-0 (Col-0) ecotype. Arabidopsis *35S*_*pro*_*:FIB2-YFP* plants were described in^23,24^. The *NRPA3*_*pro*_*:NRPA3*^*m*^*-FLAG-HA* (*nrpa3*) lines are detailed below. After sterilization, seeds were sown on 1X Murashige and Skoog (MS) medium (Duchefa Biochemie M0231), including Gamborg B5 vitamins, and supplemented with 1% (w/v) sucrose, 0.05% (w/v) 2-(N-morpholino)ethanesulfonic acid (MES), and 1% (w/v) plant agar (pH 5.7). After two days at 4 °C, the plants were grown for 15 days under a 16 h/8 h photoperiod (light/dark) in Percival growth chambers set at light intensity 180 μE·m^−2^·s^−1^ and hygrometry 55%/60% and temperature 22 °C/19 °C for light/dark, respectively.

For HS, 15-day-old seedlings were transferred to Percival chambers set at 37 °C for 4 h (during the light cycle) and 24 h (16 h light/8 h dark). For recovery experiments (R22 °C), seedlings treated for 24 h at 37 °C were returned to 22 °C (light/dark, 22 °C/19 °C) for 5 h (R22 °C 5 h) or 24 h (R22 °C 24 h).

### *NRPA3*_*pro*_*:NRPA3*^*m*^*-FLAG-HA* plant lines

Arabidopsis plants containing a T-DNA insertion in the fifth exon of *NRPA3* (At1g60850; also referred as AAC42/ATRPAC42) were obtained from The Salk Institute (Salk_088247, N588247). Heterozygous *NRPA3:nrpa3* mutant plants were then transformed with a custom-made (GeneCust, BOYNES—FRANCE) *NRPA3* gene sequence containing the ~ 1.2 kb sequence upstream from the ATG start codon and the ~ 2.1 kb sequence (introns and exons) downstream of the ATG. The *NRPA3* sequence contains three missense mutations to replace cysteines C317, C320, and C323 with serines and the sequence FLAGFLAGHAHA at the C-terminus (Figure S1).

### Nuclear protein extracts

**Figure 1:**
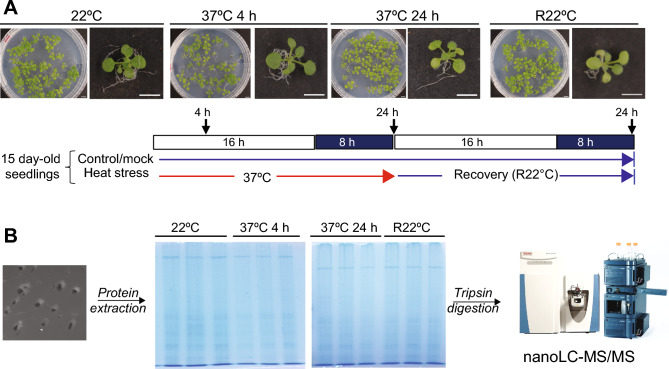
Plant growth conditions, nuclear protein extraction and nanoLC‒MSMS. (**A**) Top, 15-day-old *Arabidopsis thaliana* (Col-0) plants at 22 °C (mock), heat-treated (37 °C 4 h and 37 °C 24 h) and then returned to 22 °C for 24 h (R22 °C). The phenotypes of the seedlings are shown for each condition. Scale bar = 0.5 cm. The bottom scheme shows light/dark growth conditions and time collection points of non-treated (mock), heat-treated (37 °C 4 h and 37 °C 24 h), and recovered (R22 °C) plant samples (**B**) Left, isolated nuclei, where nucleoli appear as dark spherical bodies; middle, SDS‒PAGE and Coomassie blue staining of nuclear protein samples collected (as triplicates) at 22 °C, 37 °C 4 h, 37 °C 24 h and R22 °C; Right, nanoLC‒MS/MS analysed trypsin digestion protein samples.

For nuclear proteomic analysis, nuclear proteins were extracted from 15-day-old non-treated (22 °C), heat-treated (37 °C, 4 h and 37 °C 24 h), and recovered (R22 °C) seedlings (Fig. 1A). The nuclear protein extracts were prepared independently to generate three biological replicates per sample. Ground fine powder from approximately 2 g of seedlings was resuspended in 20 ml of cold (4 °C) 1X extraction buffer EB1X (0.5 M hexylene glycol, 0.05 M MOPS, 0.01 M MgCl_2_ × 6H_2_0) supplemented with 20 mM β-mercaptoethanol and EDTA-free Roche Protease Inhibitor. The suspension was filtered through a 60-µm nylon membrane, and 2 ml of 10% Triton X-100 was added (1% final). After 10 min of incubation, the sample was loaded onto four cushions composed of 35% (4 ml) and 80% (3 ml) Percoll and centrifuged in a JA-20 rotor at 3000 rpm (706 × *g*) for 30 min. Nuclei (white interphase between the 35% and 80% Percoll cushions) were recovered (~ 2 ml), diluted 1:2 with EB2X supplemented with 1% Triton X-100 and centrifuged for 10 min at 706 × *g*. The pellet was collected, resuspended in EB1X with 1% Triton X-100 and centrifuged for 10 min at 706 × *g*; this wash step was performed twice. The final pellet was resuspended in EB1X supplemented with 1% Triton X-100 and stored at − 20 °C. For polyacrylamide gel electrophoresis (PAGE) analysis, 1 ml of nuclear fraction was centrifuged for 10 min at 706 × *g* and resuspended in 600 µl of 4X Laemmli buffer [62.5 mM Tris–HCl pH 6.7, 2% (v/v) SDS, 10% (v/v) glycerol, 0.05% (v/v) bromophenol blue, 0.1 M DTT]. Then, 8 µl of the sample were used for proteomics analysis.

### Quantitative proteomic analysis

For liquid chromatography coupled to tandem mass spectrometry analysis (LC‒MS/MS), the nuclear protein samples were diluted in 1X Laemmli buffer supplemented with 10 mM DTT before being loaded onto an in-house 4% (v/v) acrylamide stacking gel. The gels were stained with Coomassie blue, and the stacking bands were manually excised. The proteins were then reduced, alkylated and digested overnight at 37 °C with modified trypsin in a 1:100 enzyme:protein ratio (Promega, Madison, USA). Peptides were extracted for 1 h with 80 μl of 80% acetonitrile and 0.1% formic acid before being dried and resuspended in water acidified with 0.1% formic acid prior to nanoLC‒MS/MS analysis (Fig. 1B).

The LC‒MS/MS analyses were performed using a NanoAcquity LC-system (Waters, Milford, MA, USA) coupled to a Q Exactive Plus Orbitrap (Thermo Fisher Scientific, Waltham, USA) mass spectrometer operated in Data-Dependent Acquisition mode, as previously described^24^. Peptides/proteins were identified using the Mascot search engine (version 2.5.1, MatrixScience, London, UK) against an *Arabidopsis thaliana* protein sequence database downloaded from The Arabidopsis Information Resource (TAIR) website (TAIR10 version gene model), to which common contaminants and decoy sequences were added (total of 2 × 27,534 protein entries). Identifications were validated, and label-free extracted ion chromatogram-based quantification was performed using the Proline software suite v2.0 (http://www.profiproteomics.fr/proline/)^25^. The false discovery rate was optimized to be below 1% of the PSM level using the Mascot adjusted E-value and below 1% at the protein level using the Mascot Mudpit score. Differential statistical analysis was performed on protein abundances (sum of peptide abundances) using the Prostar software suite v 1.12.11 (https://www.prostar-proteomics.org)^26^. Pairwise Limma t tests were performed. *P* value calibration was corrected using the adapted Benjamini-Hochsberg method, and FDR was set to < 1–2%. (For more details, see Supplementary Material and Method 1; Tables S1 and S2).

### RNAseq: transcriptomic analysis

For transcriptome analysis, total RNA was extracted from non-treated (22 °C), heat-treated (37 °C for 2 h, 5 h and 24 h) and recovered (R22 °C for 5 h and 24 h) seedlings. Sequencing was performed at the “Plateforme Bio-environnement” facility (UPVD, LGDP-IHPE, France) from NEBNext UltraII Directional RNA-seq libraries and using NextSeq550 (Illumina) to generate 75 bp long single reads. Raw reads were trimmed using Trimmomatic v0.39^27^. Trimmed reads were filtered for reads corresponding to mitochondrial, chloroplast and rRNA sequences using Bowtie2 v2.3.5^28^ in sensitive-local mode. Read mapping against the TAIR10 genome with Araport11 gtf file was performed using Hisat2 v2.1.0^29^. Read counting was performed using htseq-count v0.12.4^30^ in union mode and normalized to the total of mapped reads (reads per million, rpm).

Differential analysis was performed using Bioconductor R v4.1.2 package DESeq2^31^ with a false discovery rate of 0.05. *P*-values were corrected for multiple testing using the Benjamini–Hochberg rule (adjusted *P*-value). Upregulated genes were defined as having a fold change of less than two and downregulated genes as having a fold change of less than one.

### Principal component analyses, clustering and GO annotations

Principal component analysis (PCA) was performed with the prcomp() function and visualized using the autoplot() function, both from the package ggfortify v0.4.15^32^. Hierarchical clustering analysis was carried out with the hclust() function from the stats package v4.2.0 (https://stat.ethz.ch/R-manual/R-devel/library/stats/html/00Index.html) using the Pearson and complete methods as correlation and clustering methods, respectively. The gap statistic method was used to assess the optimal number of clusters using the fviz_nbclust() function from the factoextra package v1.0.7 (https://cran.r-project.org/package=factoextra). Heatmaps for cluster visualization were created with the pheatmap() function of the pheatmap package v1.0.12 (https://CRAN.R-project.org/package=pheatmap) using log2(FC) values. Line charts and boxplots were generated with the ggplot2 package v3.4.0 (https://ggplot2.tidyverse.org) using protein abundance values. All these analyses were performed in R v4.2.0 using RStudio v2022.07.2 (https://www.R-project.org). Gene Ontology (GO) annotations of protein groups were carried out with the Gene Annotation tool from TAIR (http://www.arabidopsis.org).

### Statement on experimental research on plants

The *Arabidopsis thaliana* plants (ecotype 0) comply with relevant institutional, national, and international guidelines and legislation.

## Results

### Overall nuclear proteome analysis and individual comparisons

15-day-old Arabidopsis seedlings were subjected to a short (4 h at 37 °C) or a long (24 h at 37 °C) heat stress treatment. They were then returned to optimal growth conditions (22 °C) and allowed to recover for 24 h (R22 °C). Seedlings maintained at 22 °C were used as non-treated control plants (Fig. 1A). The heat-treated seedlings (37 °C for 4 h and 37 °C for 24 h) did not show any particular phenotype during the heat treatment or recovery period compared to the non-treated plants. The integrity of the purified nuclear fraction was verified by light microscopy, and the proteins extracted were analysed by denaturing PAGE before nano LC‒MSMS (Fig. 1B).

**Figure 2:**
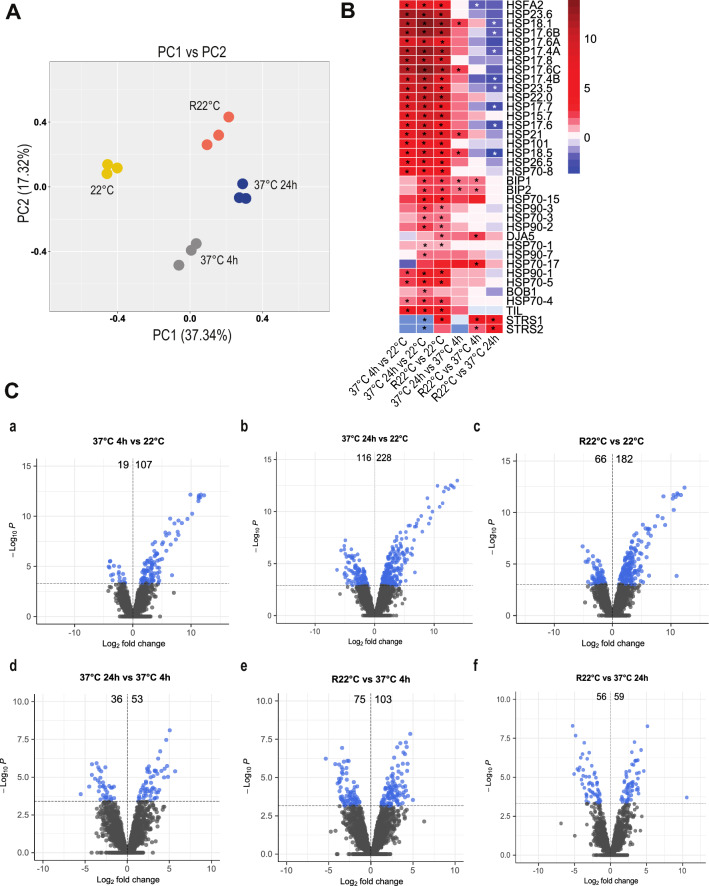
Analysis of the nuclear proteome of Arabidopsis plants under HS. (**A**) Principal component analysis (PCA) of nuclear proteins extracted from three biological replicates of non-treated (22 °C), heat-treated (37 °C for 4 h and 37 °C for 24 h) and recovered (R22 °C) plants. (**B**) Heatmaps of log_2_ FC of the heat shock factor A2 (HSFA2), heat shock proteins (HSPs), heat-induced TIL1, STRS1 and STRS2 proteins at 22 °C, 37 °C 4 h, 37 °C 24 h and R22 °C. Asterisk indicates a significant difference calculated by “Student’s t test” (**C**) Volcano plots with the differentially accumulated proteins in comparisons: 37 °C 4 h versus 22 °C (a), 37 °C 24 h versus 22 °C (b), R22 °C versus 22 °C (c), 37 °C 24 h versus 37 °C 4 h (d), R22 °C versus 37 °C 4 h (e), and R22 °C versus 37 °C 24 h (f). The number of proteins that increased (left) or decreased (right) in each comparison is indicated. Tables S1 and 2 provide the numbers of specific peptides used to generate PCA, boxplots and volcano plots.

A total of 2837, 2770, 2626 and 3064 Arabidopsis protein accessions were detected by nano LC‒MSMS in nuclear fractions from plants at 22 °C (mock), 37 °C for 4 h, 37 °C for 24 h and R22 °C, respectively (Table S1). Principal component analysis (PCA) showed that the three replicates of protein samples from non-treated, heat-treated and recovered plants grouped optimally, revealing a major contribution of the temperature change (22 °C vs. 37 °C) to the sample variability (Fig. 2A). Since the HSR involves activation of HSPs and HSFs^8,33^, accumulation of these proteins was finely examined in the nuclear proteome under different conditions. The heat shock transcription factor A2 (HSFA2) and several HSPs accumulated in the nucleus upon HS. Furthermore, the nuclear abundance of these proteins remains higher at R22 °C compared to non-treated plants (Fig. 2B).

Differential analysis was conducted to compare the four conditions (22 °C, 37 °C 4 h, 37 °C 24 h and R22 °C), and six pairwise comparisons were made: 37 °C 4 h versus 22 °C, 37 °C 24 h versus 22 °C, R22 °C versus 22 °C, 37 °C 24 h versus 37 °C 4 h, R22 °C versus 37 °C 4 h, and R22 °C versus 37 °C 24 h (Tables S2 and S3). The fold change (FC) was used as a quantitative marker of each comparison. Then, the logarithm of the FC (log_2_ FC) was calculated. For instance, when comparing 37 °C for 4 h versus 22 °C, a negative log_2_ FC indicates higher accumulation of proteins at 22 °C than at 37 °C for 4 h. On the other hand, a positive log_2_ FC corresponds to higher abundance at 37 °C for 4 h than at 22 °C. A *P* value was associated with each log_2_ FC to conclude the significance of the change in abundance (Table S3).

Considering this, 126 proteins were significantly differentially accumulated in the nucleus when comparing 37 °C for 4 h versus 22 °C. Among these 126 proteins, the abundance of 107 increased at 37 °C for 4 h, whereas the abundance of 19 decreased at 37 °C for 4 h (Fig. 2C Panel a). Similarly, 344 (228 up and 116 down), 248 (182 up and 66 down), 89 (53 up and 36 down), 178 (103 up and 75 down), and 115 (59 up and 56 down) proteins were differentially accumulated in the nucleus when comparing 37 °C 24 h versus 22 °C, R22 °C versus 22 °C, 37 °C 24 h versus 37 °C 4 h, R22 °C versus 37 °C 4 h and R22 °C versus 37 °C 24 h, respectively (Fig. 2C Panels b–f and Table S3).

Altogether, this analysis allowed identification of 522 proteins (Table S4) with nuclear abundance that significantly changed in heat-treated (37 °C for 4 h and 37 °C for 24 h) or recovered (R22 °C) plants.

### Kinetics of nuclear accumulation under HS

**Figure 3:**
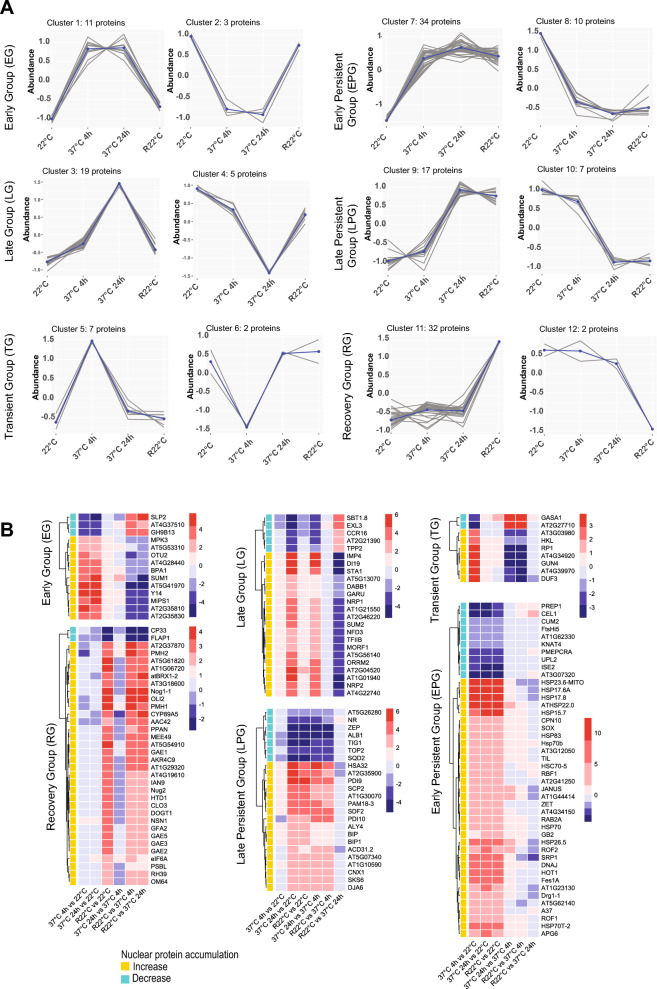
Nuclear protein accumulation under HS and recovery conditions. (**A**) Line charts show the dynamics of the 149 proteins at 22 °C, 37 °C and R22 °C in six groups: Early Group (EG), Late Group (LG), Transient Group (TG), Early Persistent Group (EPG), Late Persistent Group (LPG) and Recovery Group (RG). For each group, the left and right charts represent the amount of proteins with increasing and decreasing accumulation, respectively. The blue lines represent the overall tendency, whereas the grey lines consist of the trajectory of each member of group. (**B**) Heatmaps show the log2 FC for every protein from each group in each temperature/time comparison. The *P* values (in parenthesis) are for 37 °C 4 h versus 22 °C (0.0005); 37 °C 24 h versus 22 °C (0.001); R22 °C versus 22 °C (0.001), 37 °C 24 h versus 37 °C 4 h (0.0004), R22 °C versus 37 °C 4 h (0.0007), and R22 °C versus 37 °C 24 h (0.0005). FDR = 0.98–1.14%. Proteins with nuclear abundance increases or decreases during exposure to 37 °C are shown in yellow and blue, respectively.

The 522 proteins differentially accumulated in the nucleus were grouped into ten clusters according to their abundance (see section Principal component analyses, clustering and GO annotation; Figure S2A). In order to achieve a more reliable grouping, the significant differences between the six different comparisons (37 °C 4 h vs. 22 °C, 37 °C 24 h vs. 22 °C, R22 °C vs. 22 °C, 37 °C 24 h vs. 37 °C 4 h, R22 °C vs. 37 °C 4 h, and R22 °C vs. 37 °C 24 h) were considered to group the 522 proteins. In this way, different kinetics were built regarding accumulation of proteins in the nucleus before, during and after HS (Table S5 sheet Statistical). This approach classified 149 proteins (out of 522) into six different major groups: Early Group (EG), Late Group (LG), Transient Group (TG), Early Persistent Group (EPG), Late Persistent Group (LPG), and Recovery Group (RG; Fig. 3A and S2B).

Protein abundance in EG changed significantly after 4 h at 37 °C and remained stable up to 24 h of heat treatment. Once plants were re-exposed to 22 °C (R22 °C), the level of protein abundance was restored to that before HS (22 °C). 14 proteins belonged to EG, including eleven and three proteins with accumulation in the nucleus that increased and decreased during HS in comparison to 22 °C, respectively (Fig. 3A panel EG). Then, we performed a heatmap analysis to portray the log_2_ FC (up or down) of the six comparisons (37 °C 4 h vs. 22 °C, 37 °C 24 h vs. 22 °C, R22 °C vs. 22 °C, 37 °C 24 h vs. 37 °C 4 h, R22 °C vs. 37 °C 4 h, and R22 °C vs. 37 °C 24 h; Fig. 3B). The GO annotations reveal that the majority of the EG members are involved in “Protein Binding”, “Hydrolyse and Catalytic Activities” and “RNA Binding”, according to Molecular Function (Figure S3A panel EG). Members of EG include SMALL UBIQUITIN-RELATED MODIFIER 1 (SUM1; At4g26840), a ubiquitin-like protein involved in stress responses^34^, and MITOGEN-ACTIVATED PROTEIN KINASE 3 (MPK3; At3g45640), which participates in the MAP kinase cascade against pathogens^35^. The only nucleolar protein found in EG was RNA-binding protein Y14 (At1g51510), which is a core protein of the exon junction complex responsible for mRNA splicing^36^ (Fig. 3B panel EG, and Table S5 sheet EG).

The relative abundance of LG members in the nucleus remained significantly stable from 22 to 37 °C for 4 h but significantly changed up to 37 °C for 24 h. As in EG, the abundance of LG proteins in the nucleus returned to normal (22 °C) in the recovery phase (R22 °C). A total of 24 proteins belonged to LG: 19 and five proteins with increases and decreases in their protein abundance, respectively, at 37 °C for 24 h (Fig. 3A panel LG). GO analysis of this group mainly revealed proteins involved in “Protein Binding” and/or “RNA Binding” (Figure S3A panel LG), including the TRANSCRIPTION INITIATION FACTOR IIB-1 (TFIIB; At2g41630)^37^, the splicing factor for HSF and HSP mRNAs STABILIZED1 (STA1)^38^ and two nucleolar proteins CB-located ribonucleoprotein IMP4 (At1g63780) and CYCLOPHILIN 18-1 (At1g01940) (Fig. 3B panel LG and Table S5 sheet LG).

The relative abundance of specific proteins from TG significantly changed from 22 to 37 °C for 4 h, returning to values observed before HS at 37 °C for 24 h. TG only included nine proteins. The abundance of seven of these proteins increased at 37 °C for 4 h, while the relative abundance of the other two decreased (Fig. 3A panel TG). In addition to “Response to Chemicals”, GO analysis did not highlight any particular group regarding Molecular Function or Biological Process. However, most TG proteins are annotated as located in the “Chloroplast” (Figure S3A panel TG and Table S5 sheet TG). Among TG proteins, we found GENOMES UNCOUPLED 4 (GUN4), which is needed for synthesis of chlorophyll and plastid-nucleus communication^39,40^, and the 60S acidic ribosomal protein P2-2 (At2g27710), which also mediates protein elongation during translation^41^ (Fig. 3B panel TG and Table S5 sheet TG).

The kinetics of EPG resemble those of EG, showing a significant change in protein abundance at 37 °C for 4 h and 37 °C for 24 h compared to 22 °C. However, in contrast to EG, the change in relative abundance of these proteins persisted in the recovery phase (Fig. 3A panel EPG). Interestingly, this was the largest group, containing a total of 44 proteins. 34 proteins exhibit an abundance increase in the nucleus at 37 °C and R22 °C, whereas the abundance of 10 proteins decreases at the same time point. Several HSPs belonged to EPG, including the mitochondrial 23.6 kDa HEAT SHOCK PROTEIN (HSP23.6-MITO; At4g25200), the 17.6 kDa class I HEAT SHOCK PROTEIN 1 (HSP17.6A; At1g59860), and the peroxisomal 15.7 kDa HEAT SHOCK PROTEIN (HSP15.7; At5g37670) (Fig. 3B panel EPG and Table S5). In addition to HSPs, the TEMPERATURE-INDUCED LIPOCALIN-1 (TIL1) protein, which is involved in basal (BT) and acquired thermotolerance (AT)^42^ and translocates from the cell membrane to the cytoplasm upon salinity stress^43^, was identified (Fig. 3B panel EPG; Table S5 sheet EPG). Overall, this is consistent with the fact that the major GO annotations were “Response to Stress” and “Protein Binding” as Biological Process and Molecular Function, respectively (Figure S3B panel EPG).

As with LG, LPG corresponded to a group of proteins with significant changes in nuclear abundance at 37 °C for 24 h compared to 22 °C and 37 °C for 4 h. However, in contrast to LG, the change in protein abundance in LPG was maintained in the recovery phase (R22 °C). LPG contained 24 proteins, 17 with increasing protein abundance from 22 and 37 °C 4 h to 37 °C 24 h and seven with decreasing protein abundance (Fig. 3A panel LPG). The most common GO Molecular Function were “Protein Binding” and “Catalytic Activity”, and “Response to Stress” was the main Biological Process (Figure S3B panel LPG). Similar to EPG, proteins annotated as located in the “Chloroplast” were enriched in LPG (Figure S3B panel LPG Cellular Component). Among LPG, we found ZEAXANTHIN EPOXIDASE (ZEP; At5g67030), which is involved in ABA biosynthesis, and the protein export TRIGGER FACTOR-LIKE PROTEIN (TIG1; At5g55220). The suppressor of the transcriptional defect of Hpr1 by overexpression (THO) complex subunit ALWAYS EARLY 4 (ALY4), which participates in export of RNA molecules from the nucleus^44^, was also found in LPG (Fig. 3B panel LPG, and Table S5 sheet LPG).

Finally, in RG, the abundance of specific proteins in the nucleus did not vary significantly during HS (37 °C for 4 and 24 h), but there was a significant change during the recovery period (R22 °C). 32 of 34 proteins showed increased protein abundance at R22 °C, whereas the protein abundance of the other two decreased (Fig. 3A panel RG). The most common GO Biological Process terms were “Nucleobase-containing Compound” and “Response to Stress”; the most abundant Molecular Function terms were “RNA Binding” and “Catalytic Activity”. Interestingly, GO Cellular Component included a considerable number of proteins annotated as located in the “Nucleolus” (Figure S3B panel RG), such as the ribosome biogenesis proteins BRX1 HOMOLOGUE 2 (atBRX1-2)^45^, Arabidopsis PETER PAN-LIKE PROTEIN (APPAN)^46^, NUCLEOSTEMIN-LIKE 1 (NSN1)^47^, OLIGOCELLULA 2 (OLI2)^48^, NUCLEAR/NUCLEOLAR GTPASE 2 (ATNUG2)^49^, and the RNA Pol I subunit NRPA3^50^ (Fig. 3B panel RG and Table S5 sheet RG).

In conclusion, we identified six groups of proteins showing nuclear accumulation profile changes in an early (EG, EPG and TG) or late (LG and LPG) manner during exposure to 37 °C or after HS (RG). Although there were 29 of 149 proteins (approx. 20%) with nuclear abundance decreases during the HS and/or recovery phase, the majority (120 of 149 proteins or approx. 80%) listed in these six groups showed an increase in nuclear contents during and/or after HS.

### Identification of early-, late-, transient-like and continuous groups

**Figure 4:**
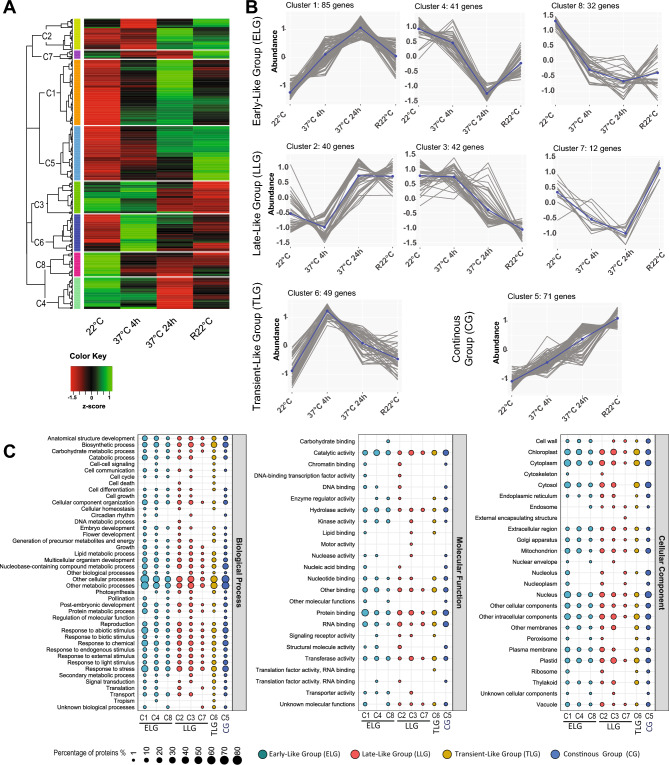
Protein accumulation in the nucleus of Early-Like (ELG), Late-Like (LLG), Transient-Like (TLG) and Continuous (CG) Groups. (**A**) Heatmap and clustering (C1–C8) of the 373 proteins differentially accumulated in response to HS but not classified in EG, LG, TG, EPG, LPG or RG. (**B**) Line charts of Clusters C1, C4 and C8, from early-like (ELG); Clusters C2, C3 and C7, from late-like (LLG); Cluster 6, from transient-like (TLG); and Cluster C5, from continuous (CG) groups are shown. Coloured squares for each cluster are given in the heatmap and line charts. (**C**) GO annotation of Clusters 1–8 in ELG (cyan), LLG (red), TLG (yellow) and CG (blue).

As mentioned above, 522 proteins were differentially accumulated in the nucleus in at least one of the six comparisons detailed above (Tables S3 and S4). However, only 149 of 522 proteins were classified as EG, EPG, LG, LPG, TG and RG (Figs. 3 and S3). Therefore, the 373 remaining proteins were reclustered based only on their protein abundances in the nucleus at 22 °C, 37 °C and R22 °C without considering significant changes for each comparison (see section Principal component analyses, clustering and GO annotation). Eight clusters (C1-C8) were generated (Fig. 4A), which were grouped according to their similarity with EG, EPG, LG, LPG, TG and RG. As a result, four additional groups were obtained: the Early-Like Group (ELG), comprising Clusters C1, C4 and C8; the Late-Like Group (LLG), comprising Clusters C2, C3 and C7; the Transient-Like Group (TLG), comprising Cluster C6; and the Continuous Group (CG), comprising Cluster C5 (Fig. 4B).

Nuclear protein accumulation at 22 °C, 37 °C and R22 °C in ELG was similar to that in EG or EPG. ELG included 158 proteins (Figs. 4B panel ELG and S4A, and Table S6 sheets ELG C1, ELG C4, and ELG C8). The main GO Molecular Function terms were “Protein Binding” and “Catalytic Activity” (Figs. 4C and S5A). Some HSPs are present in ELG, similar to EPG. In addition, nucleolar proteins were abundant, including INVOLVED IN rRNA PROCESSING 8 (IRP8)^51^, PLANT-SPECIFIC COMPONENT OF THE PRE-rRNA PROCESSING COMPLEX1 (PCP1)^52^, and the small nuclear ribonucleoprotein SMD1B involved in RNA splicing, RNA quality control, and posttranscriptional gene silencing^53^ (Figs. 4C and S5A, and Table S6 sheets ELG C1, ELG C4 and ELG C8).

For LLG, accumulation of specific proteins in the nucleus throughout the four different conditions was similar to that in LG and LPG (Fig. 4B panel LLG). We identified 94 proteins in this group (Figure S4BB and Table S6 sheets LLG C2, LLG C3 and LLG C7). The main GO Molecular Function terms were “Protein Binding”, “RNA Binding” and “Catalytic Activity” (Figs. 4C and S5B). Among them, we distinguished the nucleolar, nucleoplasm, and chromocenter-localized proteins STRESS RESPONSE SUPPRESSORS 1 and 2 (STRS1 and SRTS2, respectively). Interestingly, STRS1 and 2 are RNA helicases that attenuate the abiotic stress response^54^. LLG also contained nucleolar proteins, PONTIN/RIN1, a plant orthologue involved in telomerase assembly in the nucleolus^55^, and RNA Pol I, II, III and IV common subunit NRPB5^50^ (Table S6 sheets LLG C2, LLG C3 and LLG C7).

TLG comprised 49 proteins from Cluster C6 (Figs. 4B panel TLG and S4C, and Table S6 sheet TLG C6). This group displayed kinetics of nuclear protein accumulation similar to that of TG (Fig. 4B panel TLG). TLG contained proteins annotated as in the “Chloroplast”, “Cytosol” and “Cytoplasm” (GO Cellular Component) and various “Response to Stress” or “Biosynthetic Processes” (GO Biological Process) proteins; Figs. 4C and S5C).

CG did not resemble any of the groups described above (EG, LG, TG, EPG, LPG or RG). In CG, the abundance of 71 proteins showed continuous accumulation during the four conditions (Figs. 4B panel CG and S4D, and Table S6 sheet CG C5). The GO annotation revealed enrichment of “Response to Stress”, “Response to Chemicals” and “Response to Abiotic Stimuli” as Biological Processes. Moreover, Molecular Function terms included “Protein Binding” and “RNA Binding”; Cellular Component terms included predominantly “Nucleus” and “Cytosol/cytoplasm” (Figs. 4C and S5D). Notably, CG contained numerous nucleolar proteins involved in rRNA processing and ribosome assembly (Table S6 sheet CG C5), including RNA Pol I subunit NRPA7^50^, ARABIDOPSIS RIBOSOME PRODUCTION FACTOR 2 (ARPF2)^56^, FIBRILLARIN 1 nad 2 (FIB1 and 2, respectively)^57^, NUCLEOLIN 1 (NUC1)^58^, FK506 BINDING PROTEIN 53 (ATFKBP53)^59^, C/D snoRNP subunit NUCLEOLAR PROTEIN 56 (NOP56)^60^ and PLANT RNA HELICASE75 (PRH75)^61^. Uncharacterized nucleolar proteins, such as Gar1/Naf1 (At3g03920), a subunit of the H/ACA RNP subunit, and U3-containing 90S preribosomal complex subunit (At2g43110), were also found in CG (Table S6 sheet CG C5).

Taking these results into consideration, this analysis allowed us to identify three novel groups of proteins—ELG, LLG and TLG—displaying protein accumulation kinetics similar to those of EG, EPG, LG, LPG and TG. Furthermore, we identified an additional group (CG) showing novel kinetics of protein accumulation in the nucleus in response to and after HS.

### Proteomic versus transcriptomic analysis

**Figure 5:**
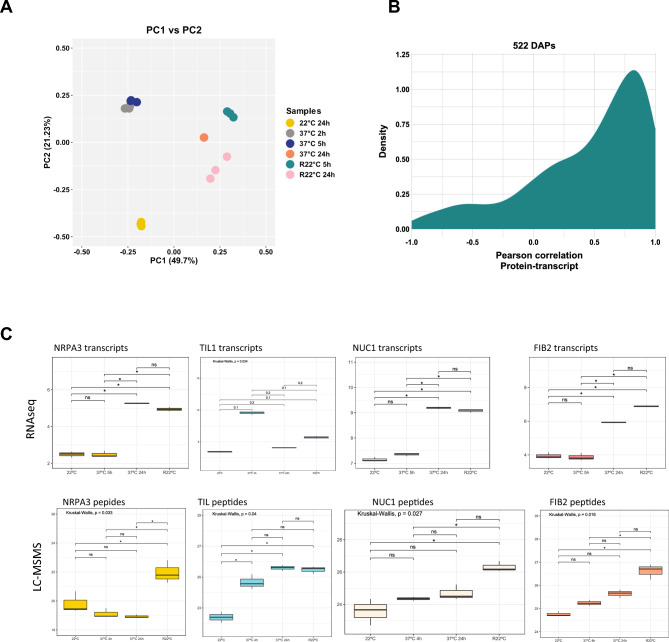
Accumulation of NRPA3, NUC1, FIB2 and TIL1 transcripts and peptides. (**A**) Principal component analysis of RNAseq replicates from Col-0 plants exposed to 22 ºC, 37 ºC (2 h, 5 h, 24 h), and R22ºC (5 h and 24 h). (**B**) Density plot of Pearson correlation coefficients between protein (22ºC, 37ºC 5 h, 37ºC 24 h and R22ºC 24 h) and transcript (22ºC, 37ºC 4 h, 37ºC 24 h and R22ºC 24 h) abundance for 522 differentially abundant proteins (DAPs) (top panel and Table S8). (**C**) Box plots of the number of specific NRPA3 (At1g60850), TIL1 (At5g58070), NUC1 (At1g48920) and FIB2 (At4g25630) transcripts (Table S8) and peptides (Table S2) at 22 °C, 37 °C (5 h and 24 h) and R22 °C. Asterisks indicate t-student *P*-values: * ≤ 0.05, ns, not significant.

We investigated whether the changes in nuclear accumulation observed during HS and recovery periods were due to changes in the amount of total cellular protein and/or to changes in mRNA levels. We performed RNAseq of non-treated (22 °C), heat-treated (37 °C) and recovered (R22 °C) plants. RNAseq was performed on three biological samples and showed reproducible differences between temperature treatment conditions (Fig. 5A and Table S7).

Comparative (proteomic and transcriptomic) analysis of the 522 differentially accumulated proteins in the nucleus revealed differences in protein abundance and the accumulation of corresponding transcripts during and after HS. These changes in protein and transcript levels can be positively or negatively correlated (Fig. 5B and Table S8). Most of them are positively correlated (a proportion of about 59% with a Pearson correlation between 0.5 and 1) in all comparisons between different treatments. We also observed a proportion of genes that are not correlated (~ 33% of genes) and a small group with a negative correlation (~ 7% of genes). These correlations are similar for each treatment comparison (Figure S6).

**Figure 6:**
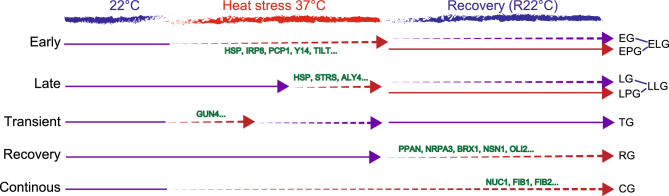
Illustration of the five tendencies of protein accumulation upon heat stress (37 °C) and recovery (R22 °C) conditions. Early (EG, EPG and ELG), late (LG, LPG and LLG), transient (TG and TLG), recovery (RG) and continuous (CG) tendencies are represented. Arrows with continuous lines indicate no changes in protein accumulation, and arrows with dashed lines indicate significant changes (increase or decrease) in protein abundance. Red arrows show changes in protein abundance compared to 22 °C (before stress), while purple arrows show protein abundances similar to 22 °C.

Next we focused this comparative analysis on nucleolar proteins belonging to RG and CG. This is mainly due to the significant abundance of nucleolar proteins in these two groups (Figures S3B panel RG and S5D). Another reason is that the most prominent and multifunctional nuclear structure, is disorganized during HS and reorganises during the recovery period^19^. Among the nucleolar proteins we selected the subunits of RNA Pol I NRPA3 (AAC42/ATRPAC42)^50^ detected in RG, and NUC1^58,62,63^ and FIB2^57^, both detected in CG (Tables S5 sheet RG and S6 sheet CG). We also examined the protein accumulation of TIL1, which is involved in thermotolerance^42^ and detected in EPG. Consistent with the LC–MSMS results (Fig. 6); Western blot analysis also shows accumulation of these proteins in response to heat stress and recovery conditions (Figure S7 and Table S9).

RNAseq analysis revealed an accumulation of NRPA3, NUC1 and FIB2 transcripts in response to prolonged heat stress (37 °C for 24 h) and during the recovery conditions (R22 °C). In contrast, TIL1 transcripts accumulate transiently after 4 h at 37 °C (Fig. 5C). Nevertheless, the accumulation of NRPA3, NUC1 and FIB2 transcripts is mainly correlated with peptide accumulation during the recovery conditions whereas the accumulation of TIL1 transcripts and peptides are only correlated after 4 h at 37 °C (Fig. 5C, L-MSMS panel). Overall, Pearson correlation values (peptides v/s transcripts) indicate a positive correlation only for NUC1 (0.78), while no correlation is observed for NRPA3 (0.31), FIB2 (0.49) and TIL1 (0.32).

## Discussion

In this study, we performed a quantitative nanoLC‒MSMS analysis of the nuclear proteome of Arabidopsis at 22 °C, 37 °C for 4 h, 37 °C for 24 h and R22 °C. Our analysis revealed pronounced and relatively rapid changes in the proteomic profile of the nucleus upon HS. To our knowledge, this is the first label-free quantitative analysis of the Arabidopsis nuclear proteome under sustained HS. The proteome of whole-cell extracts of Arabidopsis plants exposed to extreme and short HS stress leading to seedling survival or death has been examined^64^. Other proteomic studies using whole-cell extracts have also been carried out under HS in other plants, including spinach^65^, tomato^66^ and *Clematis florida*^67^.

In our investigation, ~ 20% of proteins detected by nanoLC‒MSMS (522 of 2629) exhibited differential nuclear accumulation during HS and/or the recovery stage (Fig. 6). Notably, in response to HS and/or recovery conditions, most proteins (366 proteins) increased in nuclear accumulation, whereas the others (156 proteins) showed decreases. Consequently, these changes might induce or inhibit nuclear protein activities in response to HS. Remarkably, most of these proteins are related to diverse stress responses, including light intensity and redox (Figure S8), which are interrelated with HS^68,69^.

Most HSPs were found in EPG or ELG, as they strongly accumulated in the nucleus upon HS and remained relatively stable during recovery. Furthermore, other proteins that accumulated in the nucleus under HS were detected, including STRS2^54^ and TIL1^42^, which are involved in HSR and thermotolerance, respectively. In addition to HSFs, HSPs, and other heat-induced proteins, we did not identify robust groups of proteins associated with specific Molecular Functions and/or Biological Processes according to their GO annotations. Nevertheless, the categories “Response to Stress” (Biological Process) and “Protein Binding”, “RNA Binding” and “Catalytic Activity” (Molecular Function) were abundant in most of the groups (EG/ELG, LG/LLG, TG/TLG, EPG and CG).

Of the 522 differentially accumulated proteins in the nucleus, 233 contain a predicted nuclear localisation signal (NLS), 198 are annotated as nuclear, and only 5 as transcription factors, these include HSFA2, WRKY transcription factor NUCLEAR FUSION DEFECTIVE 1 (NFD1), the Arabidopsis NAC domain-containing protein 2 (ANAC002), Homeobox KNOTTED1-LIKE HOMEOBOX GENE 4 (KNAT4) and the histone deacetylase 3 (HDA3) (Table S4). . Besides, several are annotated as chloroplast-located and/or functionally related to the chloroplast, including AGY1^70^ and GUN4^39^. Interestingly, both AGY1 (LLG) and GUN4 (TG) trigger retrograde signalling, indicating that HS affects the information flux from plastids to the nucleus. In agreement, retrograde signalling GUN4 contributes to acquisition of basal thermotolerance (BT) in Arabidopsis^71^. In contrast to AGY1 and GUN4, nuclear abundance of the ribonuclease III family protein RNC1 (At4G37510) rapidly decreased upon HS (EG). RNC1 lacks endonuclease activity, but its RNA binding activity is needed for splicing in the chloroplast^72^. The potential role of RNC1 in the nucleus remains uncertain. Only a few differentially accumulated proteins in our analysis are annotated as mitochondrial proteins. These included two HSPs (EPG) and at least four RNA binding proteins (two mRNA editing activities in LG and two helicases in RG). Whether these proteins are involved in a mitochondrial retrograde pathway remains unknown. However, it has been reported that specific mitochondrial unfolded and degraded proteins enter the nucleus and regulate transcription of genes related to mitochondrial protein homeostasis under stress conditions^73^. Moreover, other cytoplasmic proteins accumulated in the nucleus upon HS or during the recovery period, indicating that cytosolic and/or organelle activities are inhibited or activated. These include RNA and/or protein binding activities and other metabolic processes. Further and precise characterization of these proteins should be carried out for a better understanding of the molecular clues and mechanisms controlling protein nuclear translocation in response to HS.

Out of the 522 differentially accumulated proteins, 149 were assigned to EG, LG, TG, EPG, LPG or RG; the other proteins (373 accessions) were classified in ELG, LLG, TLG or CG. All groups, except for RG, included proteins with changes in nuclear abundance upon HS, either in an early, transient, or persistent manner. In contrast, proteins included in RG showed accumulation profile changes exclusively after HS, in other words, during the recovery period. Notably, we observed that this recovery period (R22 °C) allowed for partial reestablishment of the nuclear proteomic profile. The existence of EPG/ELG, LPG/LLG, RG and CG portrays significant accumulation of proteins at R22 °C. In addition, these groups were more abundant in comparison to EG, LG or TG. This suggests that the “recovery” phase established as 22 °C for 24 h after HS did not fully restore levels observed during standard conditions (22 °C). This was also evident in PCA (Fig. 2A), where the R22 °C and 37 °C 4 h replicates were practically equidistant from the 22 °C replicates. Thus, a longer recovery period, i.e., 22 °C for 48 or 60 h after HS, may achieve substantial recovery. Overall, the amount of proteins exhibiting significant nuclear accumulation in the new recovery phase must decrease dramatically.

Comparative proteomic and transcriptomic analysis revealed that an increase or decrease in relative protein abundance in the nucleus may or may not be associated with a change in the transcript levels. Therefore, observed changes in nuclear protein abundance of specific proteins could be due to multiple factors, including protein translocation and/or changes in protein stability and/or gene expression in response to heat stress. Similarly, differences between proteomic and transcriptomic data are also observed in different Arabidopsis tissues and organs^74^.

Notably, several nucleolar proteins were found in RG, including NRPA3^50^, BRX1^45^, NSN1^47^, OLI2^48^, APPAN^46^, and NUCLEAR/NUCLEOLAR GTPASE 2 (ATNUG2)^49^. Similarly, accumulation of other nucleolar proteins, including NUC1^58,62,63^ and FIB2^57^, was observed during the recovery period as part of CG. All these proteins are involved in rRNA synthesis and/or ribosome biogenesis. As mentioned above, the nucleolus is the most prominent subnuclear structure, and its assembly results in the transcription and processing of rRNA and assembly of ribosome particles^75^. In Arabidopsis, pre-rRNAs transcription and processing, ribosome profiles, and functional nucleolar structures are disrupted upon HS and restored gradually after the plants are returned to optimal growth conditions^19^. Interestingly, heat stress treatment does not affect the localisation of FIB2, but it is reorganised in the "disrupted" nucleolus^74^.

Therefore, increased nuclear accumulation of NUC1, FIB2, and other ribosome biogenesis factors (RBFs) during the recovery period may be required to fully restore nucleolar assembly and activity after HS. This phenomenon, which has been termed “nucleolar recovery”, may be comparable to HSR. As with HSPs or HSFs during HSR, there is strong accumulation of RBFs at R22 °C to promote this nucleolar recovery. Subsequently, this increase of RBFs aims to restart ribosome biogenesis. It would be necessary to examine the nuclear abundance of these RBFs at a shorter time point in the recovery phase (22 °C for 4 h after HS) to assess how rapid this response is. The function of the nucleolus is strongly related to its tripartite structure^76^. Thus, the spike in RBFs during the recovery phase may occur to re-establish the regular nucleolar architecture after HS. Remarkably, the nucleolus is a multiphase liquid condensate^21^ that requires FIBRILLARIN and the scaffold proteins of the granular component NUCLEOPHOSMIN (NPM1). Both proteins contain IDR (Intrinsically Disordered Region) and GAR (Gly–Arg-rich domain) domains. To our knowledge, an NPM1 orthologue has not been reported in Arabidopsis; however, NUC1 also contains IDRs, GAR domains, and acidic stretches^63^, which might contribute to the formation of condensates governed by liquid–liquid phase separation (LLPS) (Figure S9). Furthermore, GAR1 accumulates in the nucleus during the recovery period and might also drive LLPS^77^. In addition to protein composition, protein concentration, protein posttranslational modifications, and the presence of an RNA seed to promote nucleation are essential to drive LLPS^77^. In this context, increasing the level of IDR/GAR-containing nucleolar proteins during the recovery period might contribute to effectively reassembling the nucleolus after HS.

### Supplementary Information

Supplementary Information 1.

Supplementary Information 2.

Supplementary Information 3.

Supplementary Information 4.

Supplementary Information 5.

Supplementary Information 6.

Supplementary Information 7.

Supplementary Information 8.

Supplementary Information 9.

Supplementary Information 10.

Supplementary Information 11.

Supplementary Information 12.

Supplementary Information 13.

Supplementary Information 14.

Supplementary Information 15.

Supplementary Information 16.

Supplementary Information 17.

Supplementary Information 18.

Supplementary Information 19.

Supplementary Information 20.

Supplementary Information 21.

Supplementary Information 22.

Supplementary Information 23.

Supplementary Information 24.

Supplementary Information 25.

Supplementary Information 26.

Supplementary Information 27.

Supplementary Information 28.

Supplementary Information 29.

## Data Availability

The mass spectrometry proteomics data have been deposited in the ProteomeXchange Consortium via the PRIDE^78^ partner repository with the dataset identifiers PXD045038 and 10.6019/PXD045038https://www.ebi.ac.uk/pride/login. All RNAseq data have been submitted to the Sequence Read Archive (SRA): BioProject PRJNA972651.
