# Supplementary figures and images for: Proteomic profiling of Arabidopsis nuclei reveals distinct protein accumulation kinetics upon heat stress
Source: Sci Rep. 2024 Aug 14;14:18914. doi: 10.1038/s41598-024-65558-4 (PMC11324732; doi:10.1038/s41598-024-65558-4)

Figure S1

**A**

NRPA3 (At1g60850)

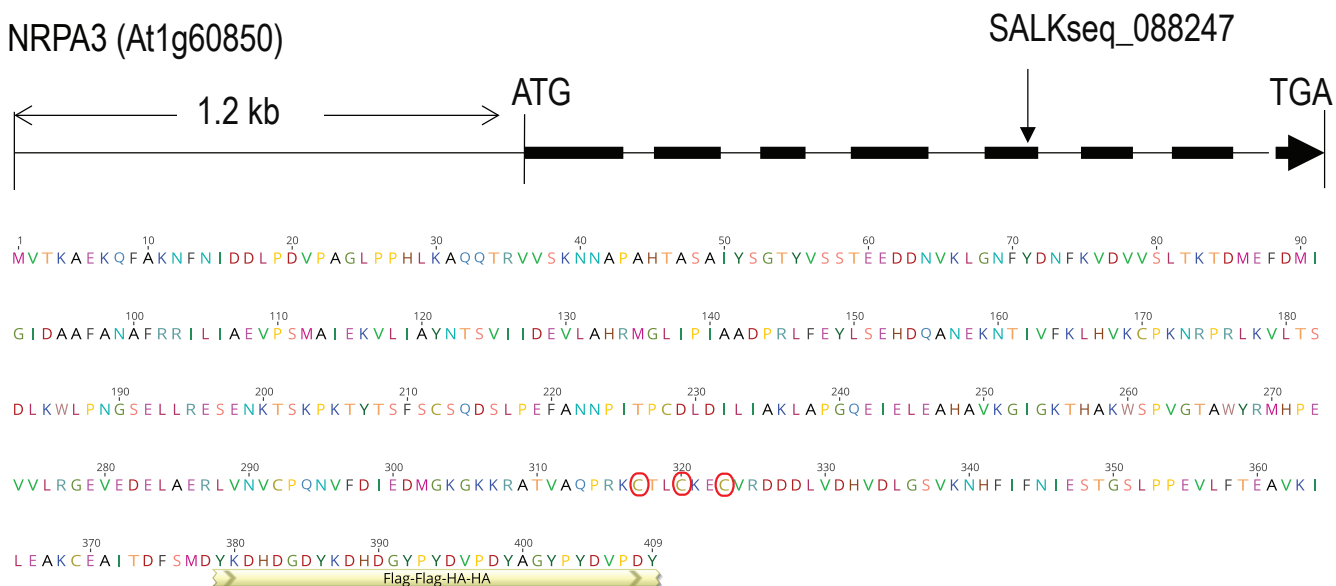**B**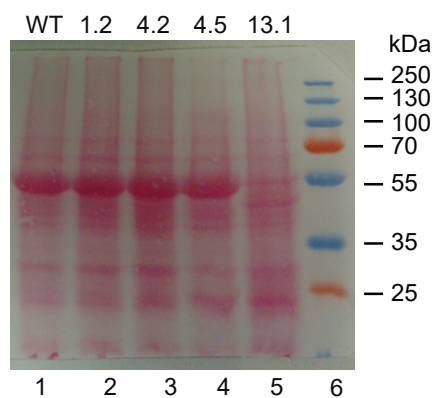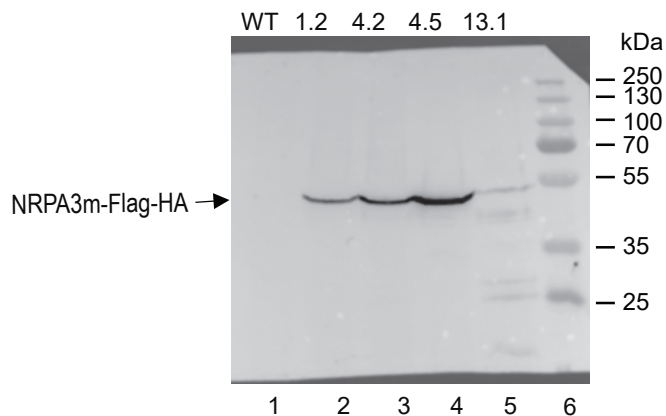

Supplement: Supplementary file 1 — Supplementary Information 1. [file 41598_2024_65558_MOESM1_ESM.pdf]

Figure S2

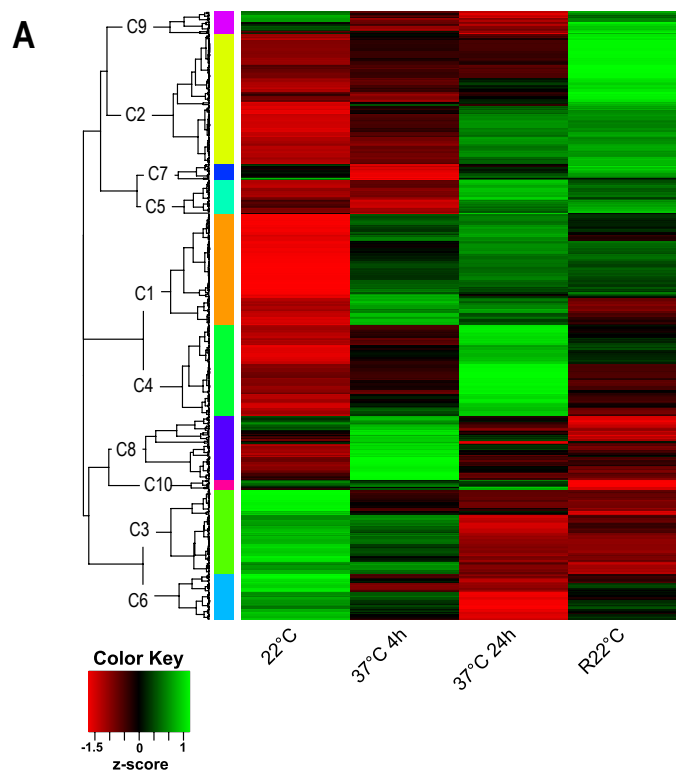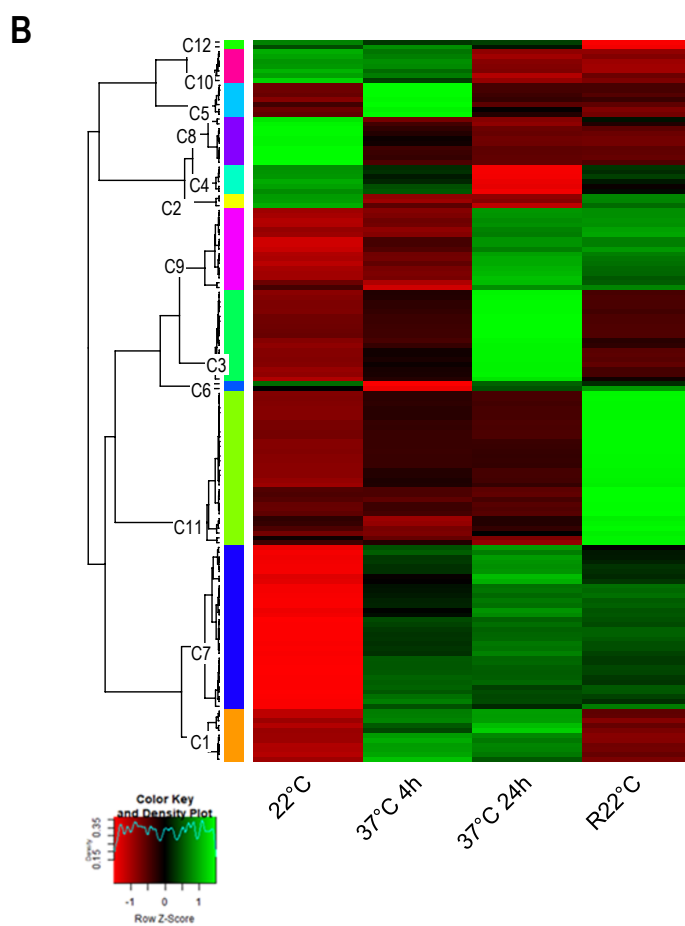

Supplement: Supplementary file 2 — Supplementary Information 2. [file 41598_2024_65558_MOESM2_ESM.pdf]

Figure S3A

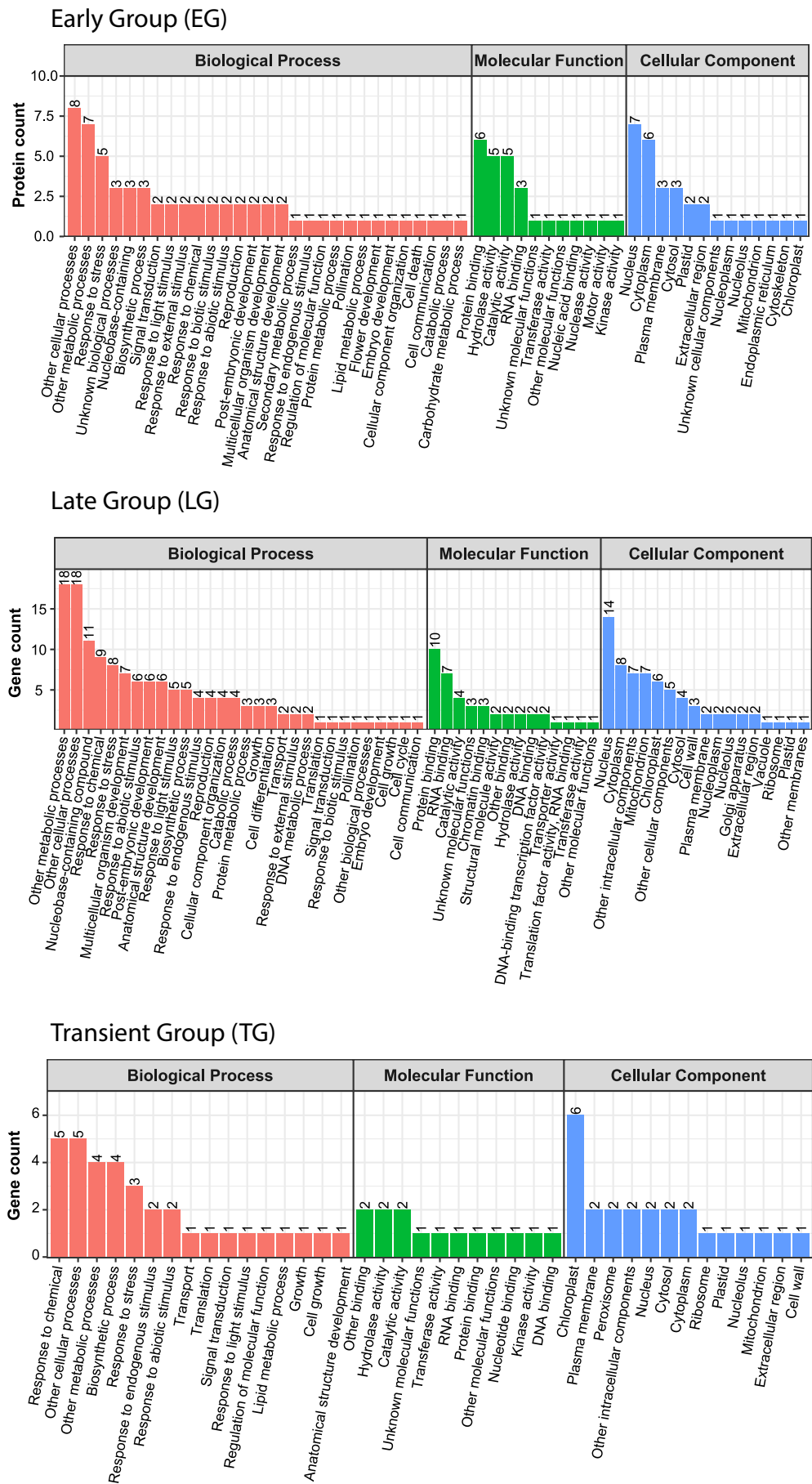

Supplement: Supplementary file 3 — Supplementary Information 3. [file 41598_2024_65558_MOESM3_ESM.pdf]

## Early Persistent Group (EPG)

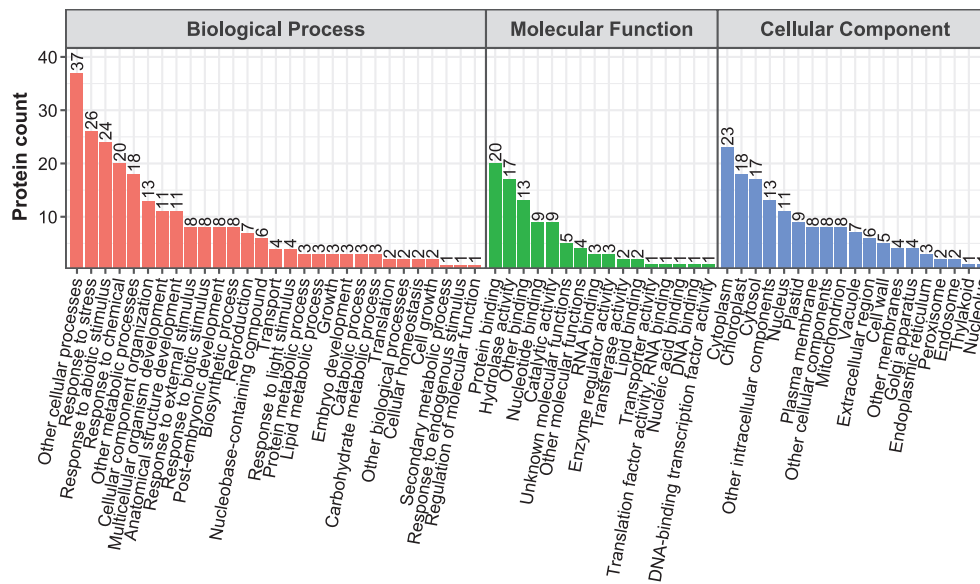

## Late Persistent Group (LPG)

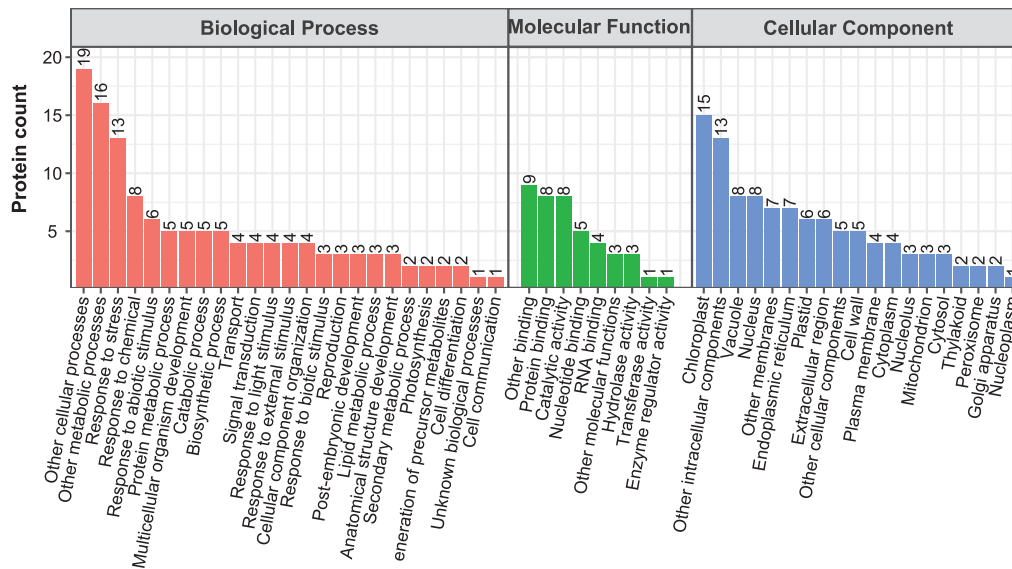

## Recovery Group (RG)

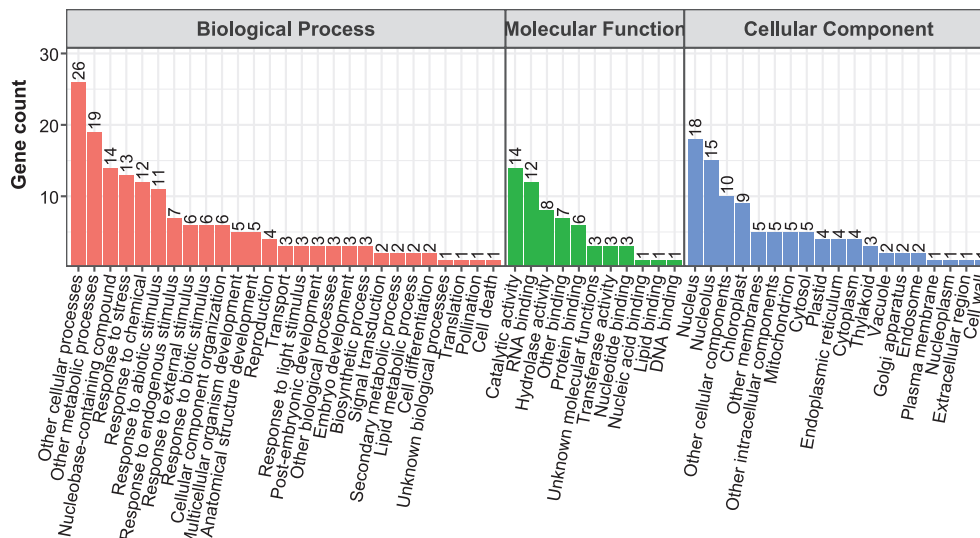

Supplement: Supplementary file 4 — Supplementary Information 4. [file 41598_2024_65558_MOESM4_ESM.pdf]

Figure S4A

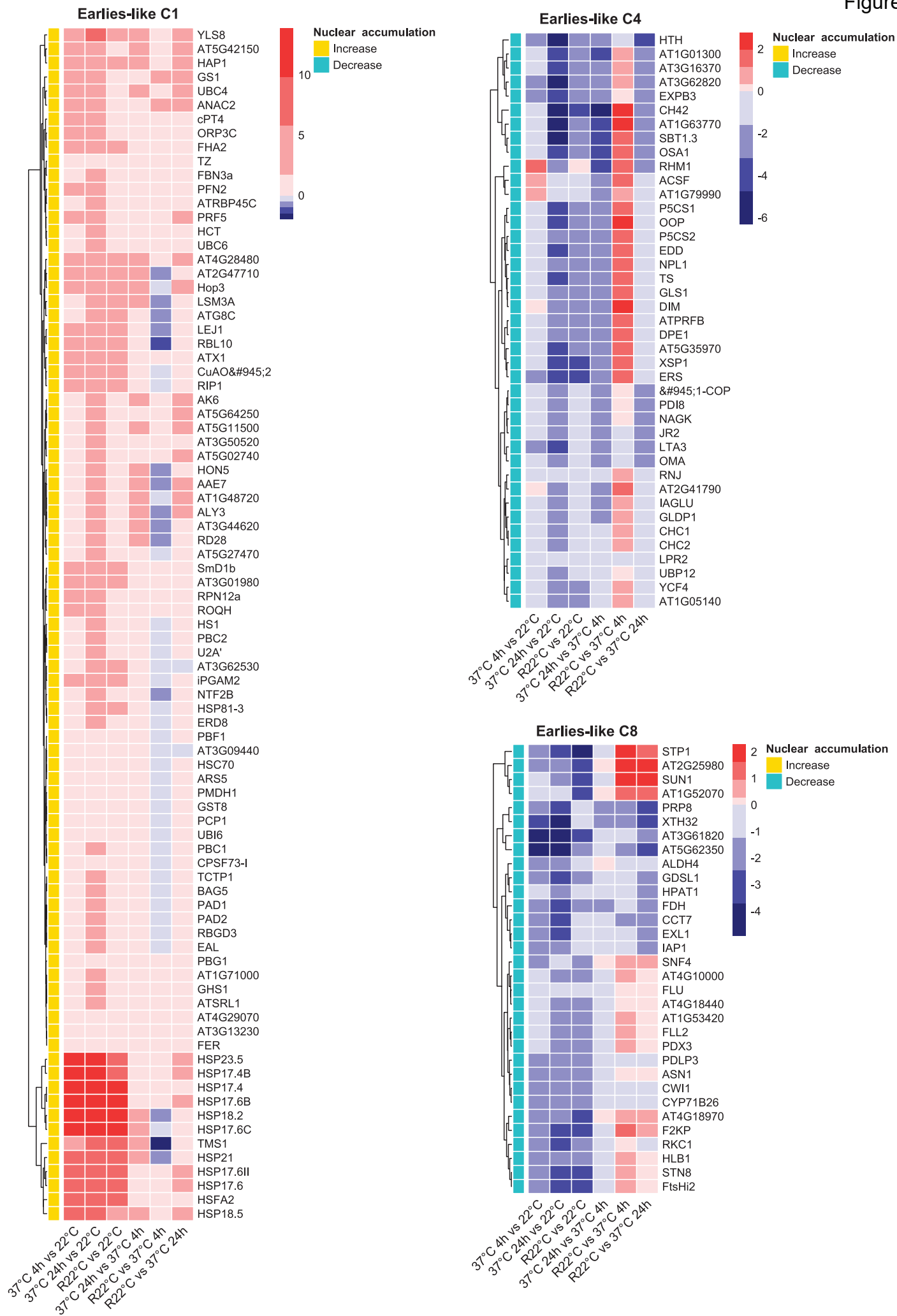

Supplement: Supplementary file 5 — Supplementary Information 5. [file 41598_2024_65558_MOESM5_ESM.pdf]

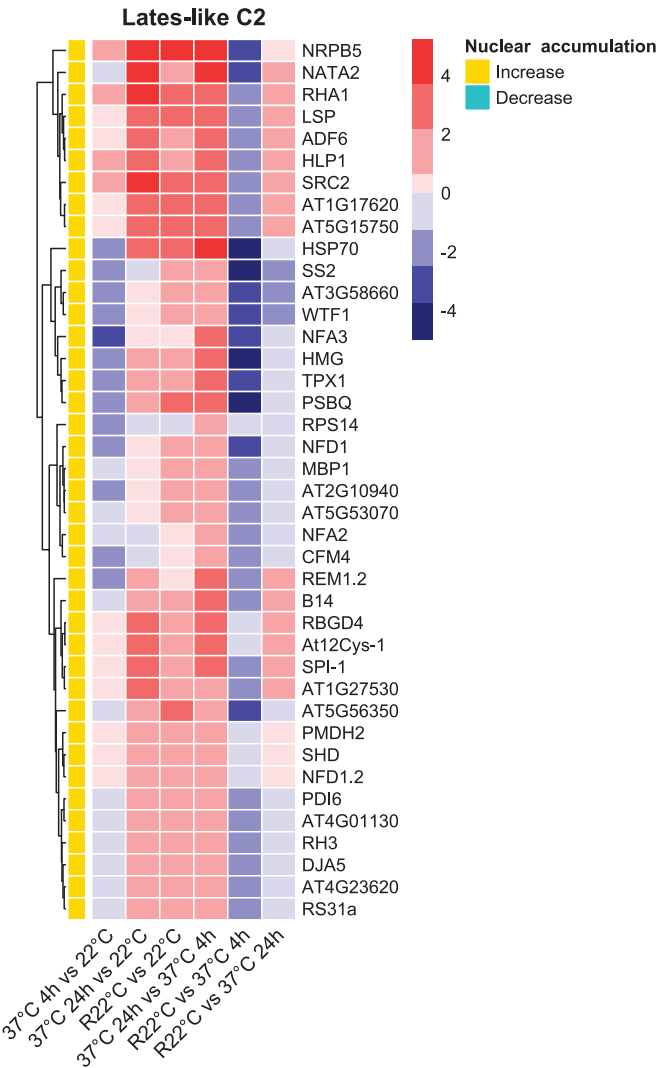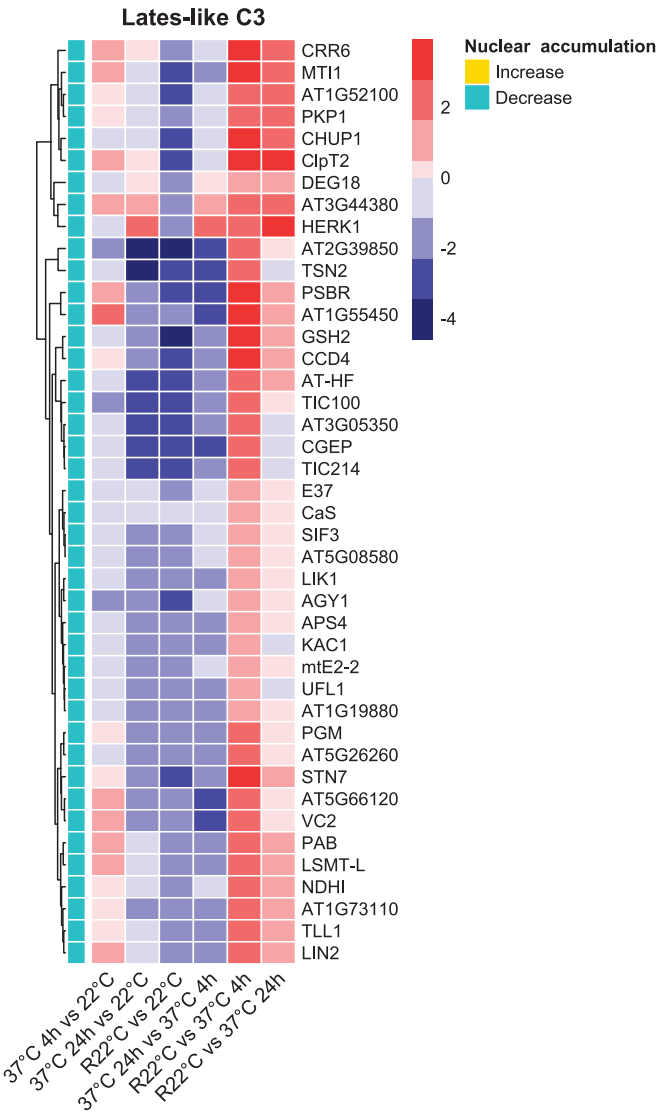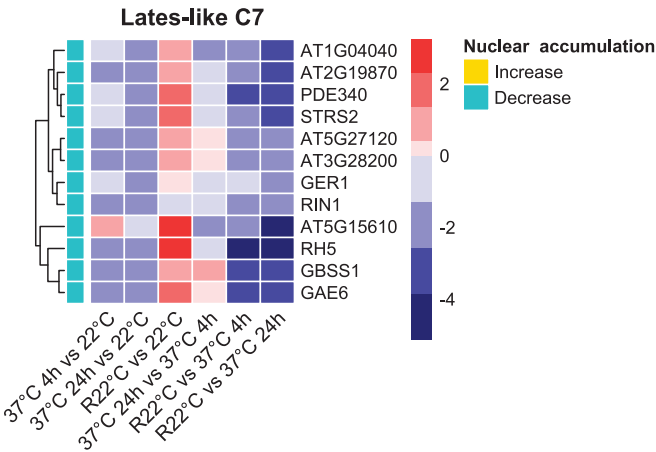

Supplement: Supplementary file 6 — Supplementary Information 6. [file 41598_2024_65558_MOESM6_ESM.pdf]

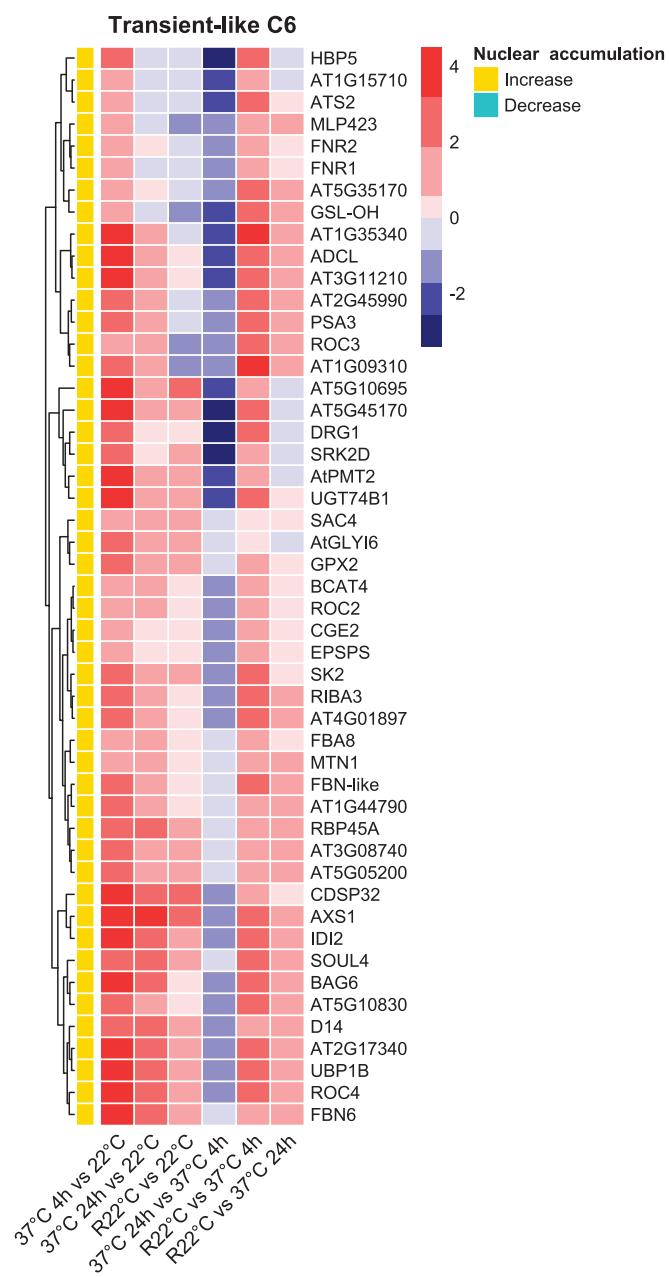

Supplement: Supplementary file 7 — Supplementary Information 7. [file 41598_2024_65558_MOESM7_ESM.pdf]

Figure S4D

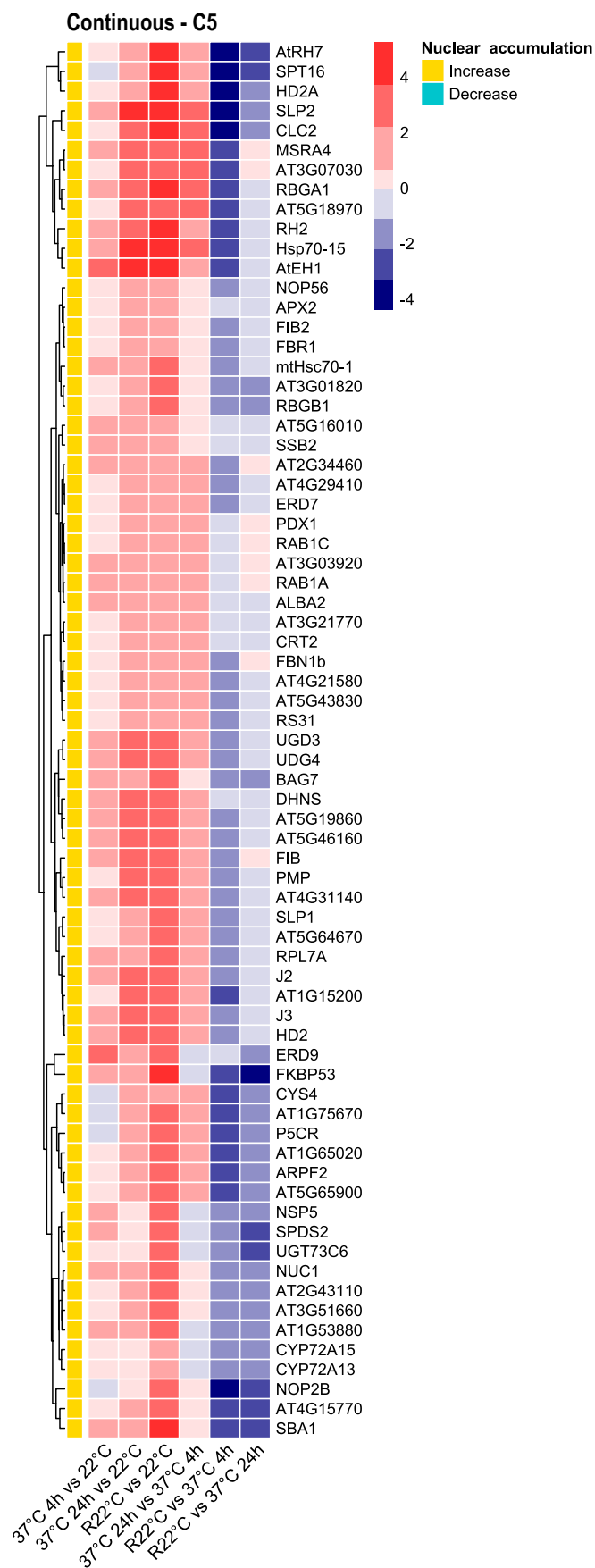

Supplement: Supplementary file 8 — Supplementary Information 8. [file 41598_2024_65558_MOESM8_ESM.pdf]

Late-Like Group (LLG)

Cluster 2

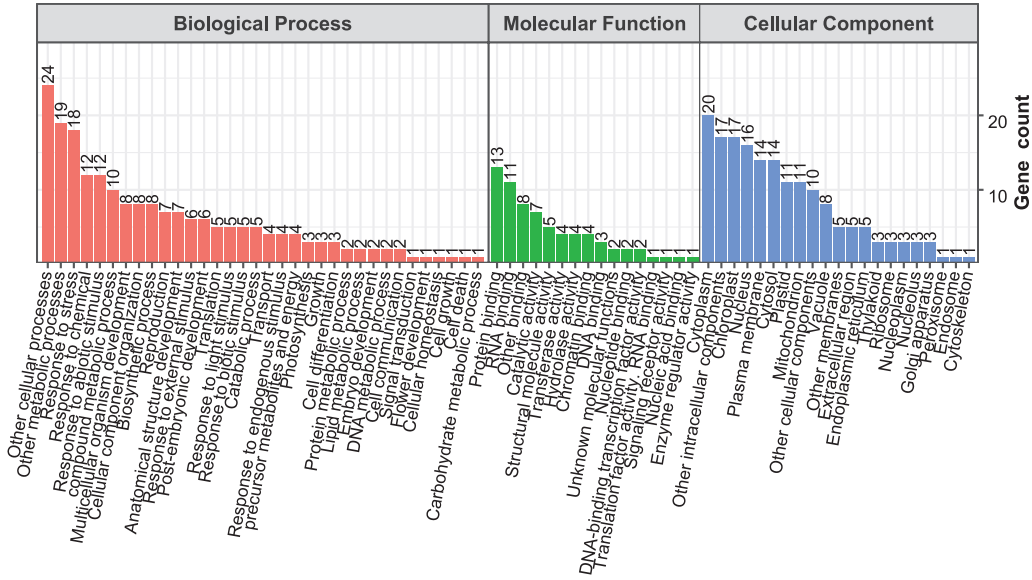

Cluster 3

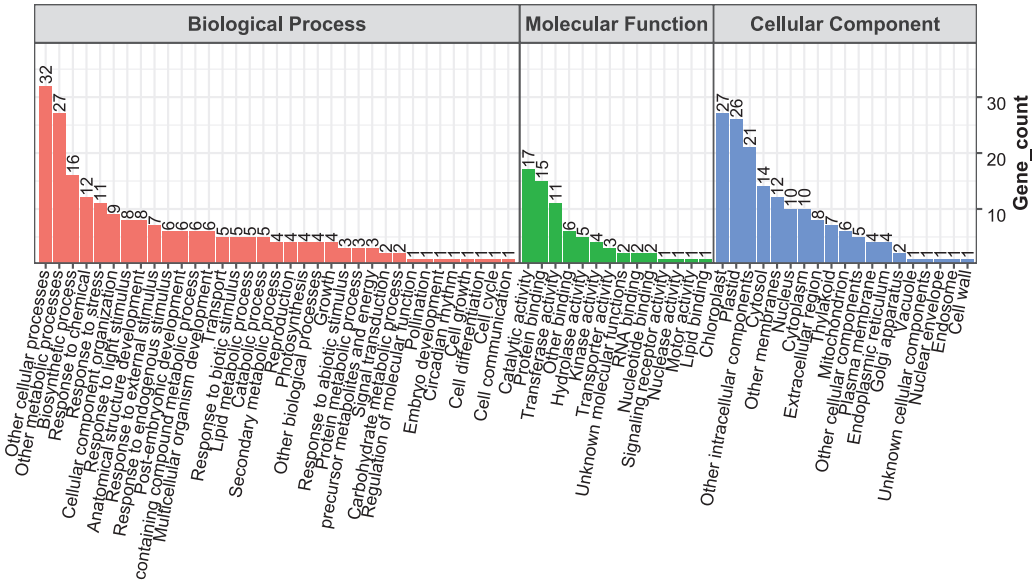

Cluster 7

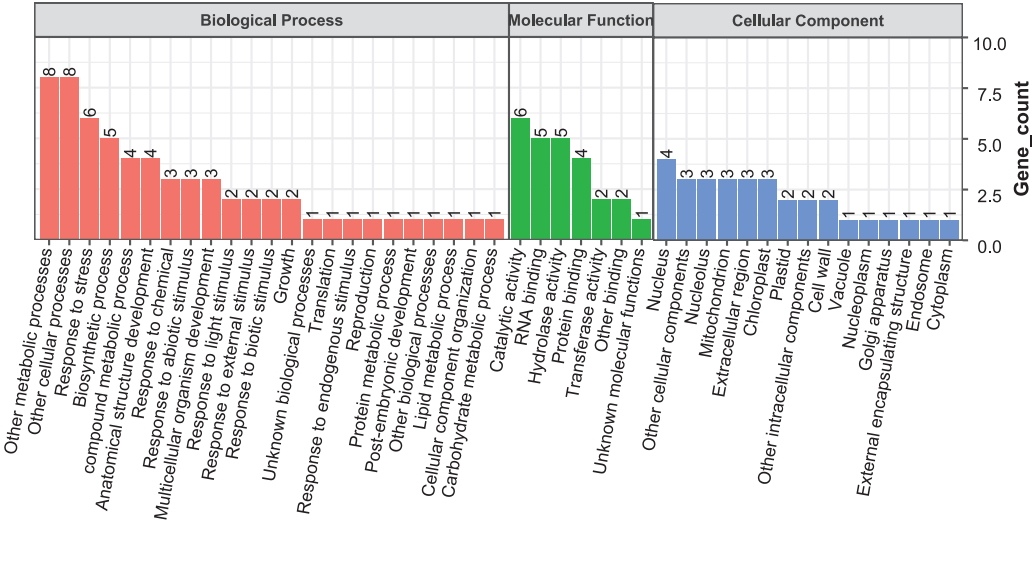

Supplement: Supplementary file 10 — Supplementary Information 10. [file 41598_2024_65558_MOESM10_ESM.pdf]

Cluster 6

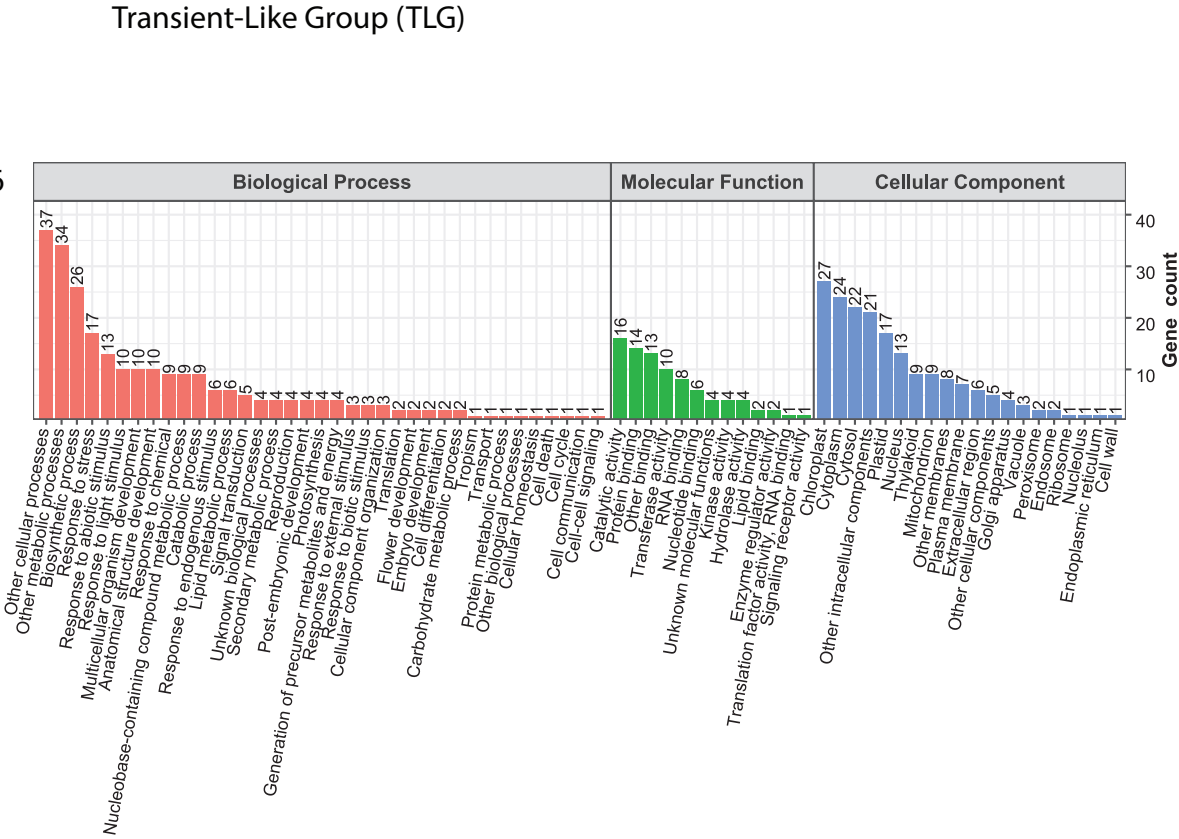

Supplement: Supplementary file 11 — Supplementary Information 11. [file 41598_2024_65558_MOESM11_ESM.pdf]

Continous Group (CG)

Cluster 5

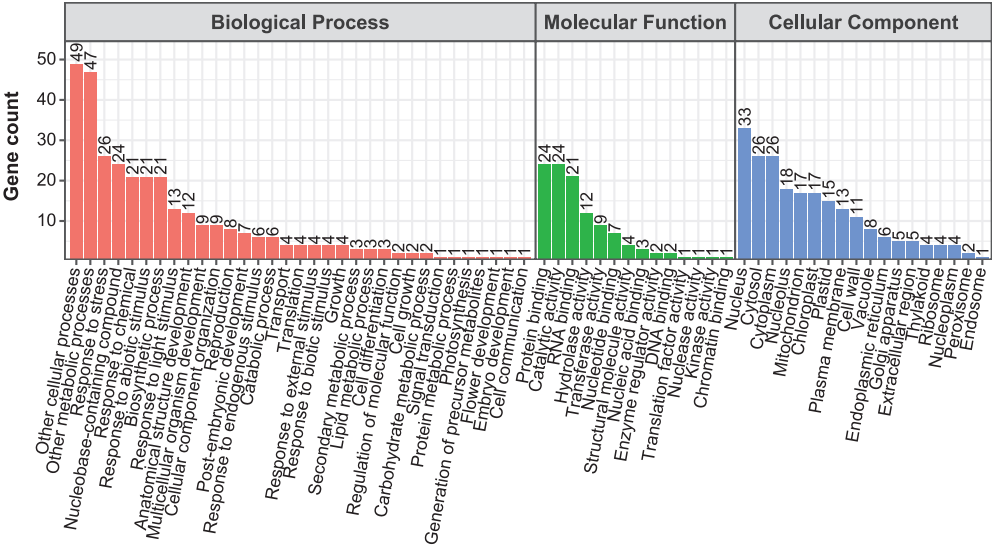

Supplement: Supplementary file 12 — Supplementary Information 12. [file 41598_2024_65558_MOESM12_ESM.pdf]

Figure S6

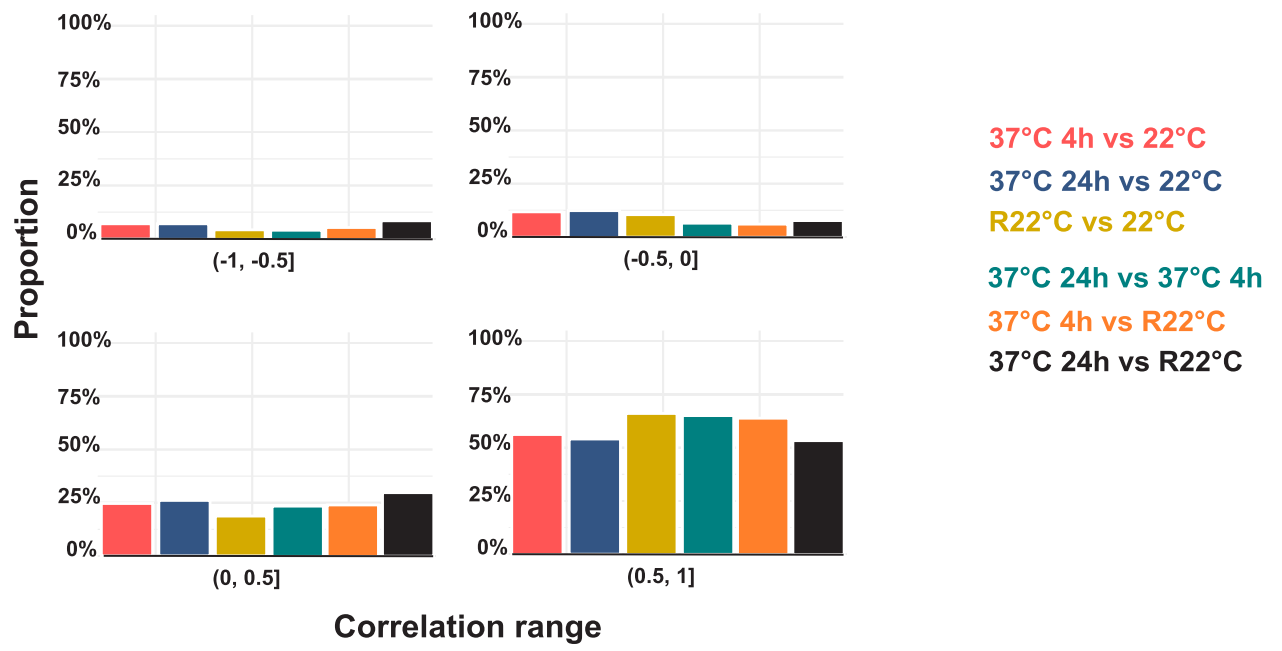

Supplement: Supplementary file 13 — Supplementary Information 13. [file 41598_2024_65558_MOESM13_ESM.pdf]

Figure S7

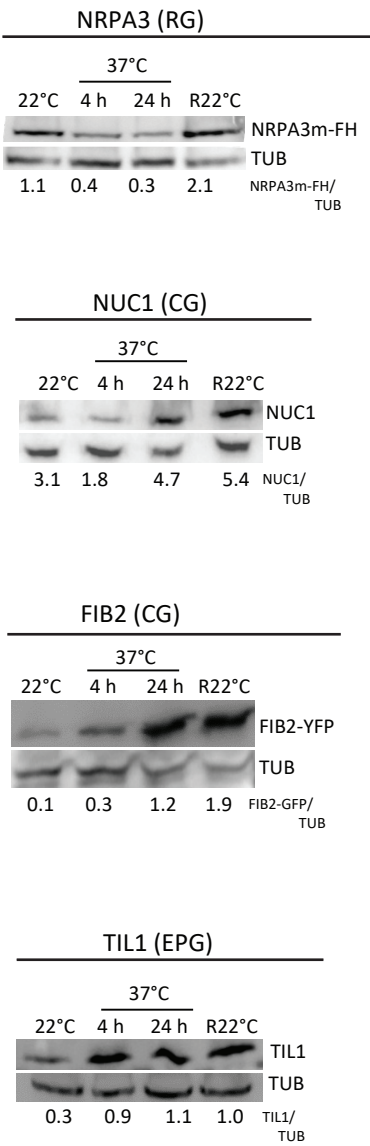

Supplement: Supplementary file 14 — Supplementary Information 14. [file 41598_2024_65558_MOESM14_ESM.pdf]

Figure S8

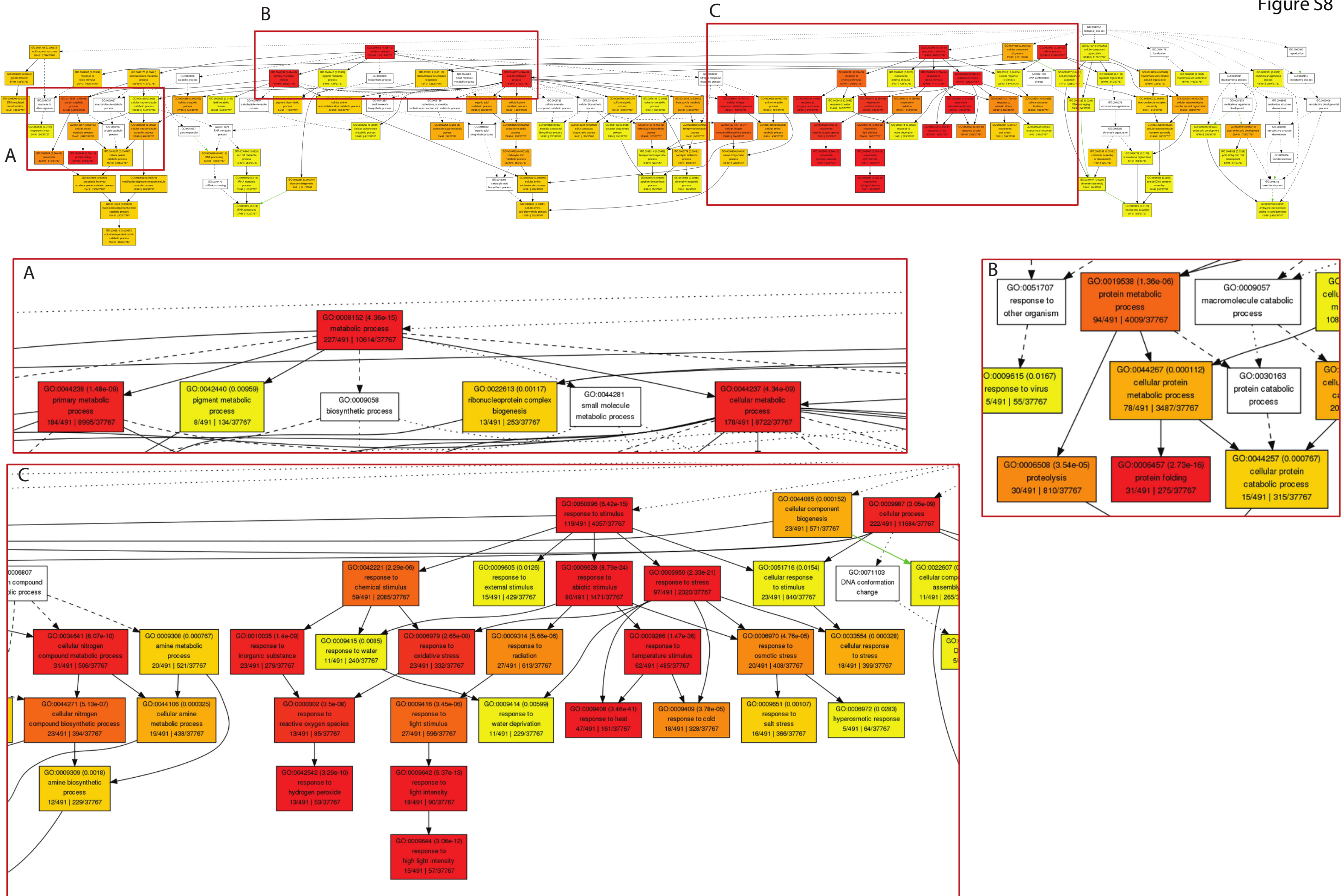

Supplement: Supplementary file 15 — Supplementary Information 15. [file 41598_2024_65558_MOESM15_ESM.pdf]

Figure S9

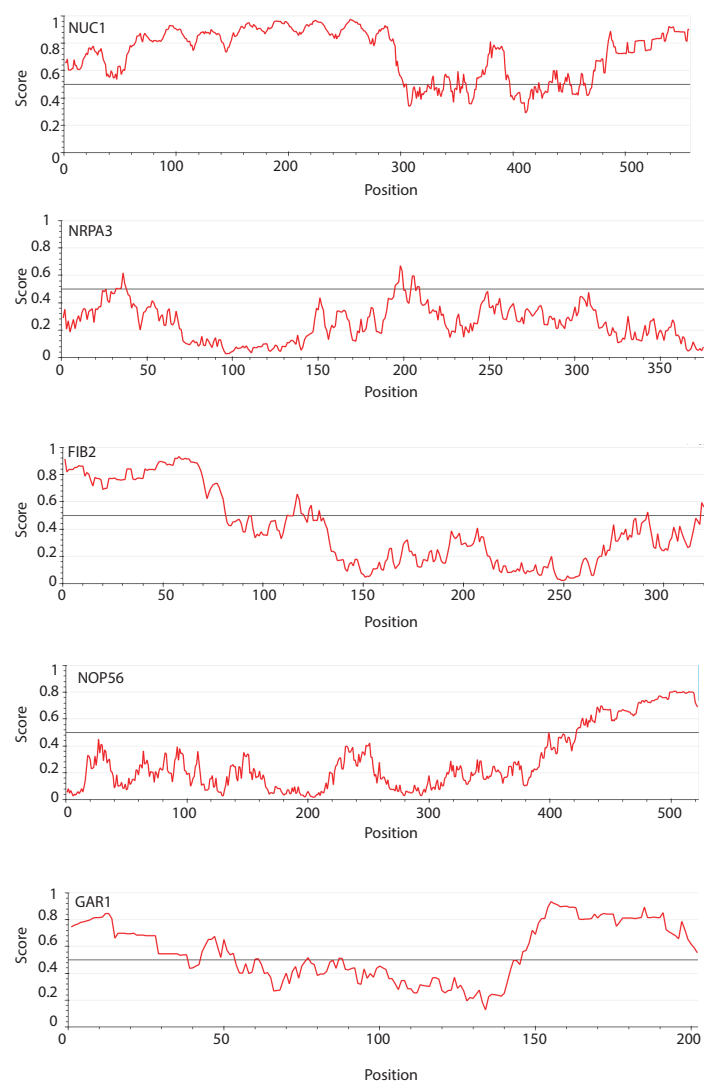

Supplement: Supplementary file 16 — Supplementary Information 16. [file 41598_2024_65558_MOESM16_ESM.pdf]

Supplememental Information: Full length gels shown in main Figure 1B

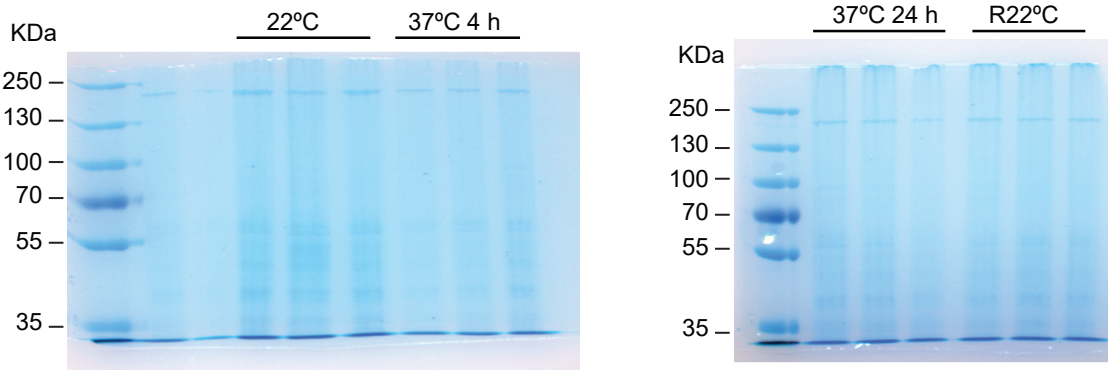

Supplement: Supplementary file 19 — Supplementary Information 19. [file 41598_2024_65558_MOESM19_ESM.pdf]

Supplemental Information: Full Western blots shown in Figure S7.

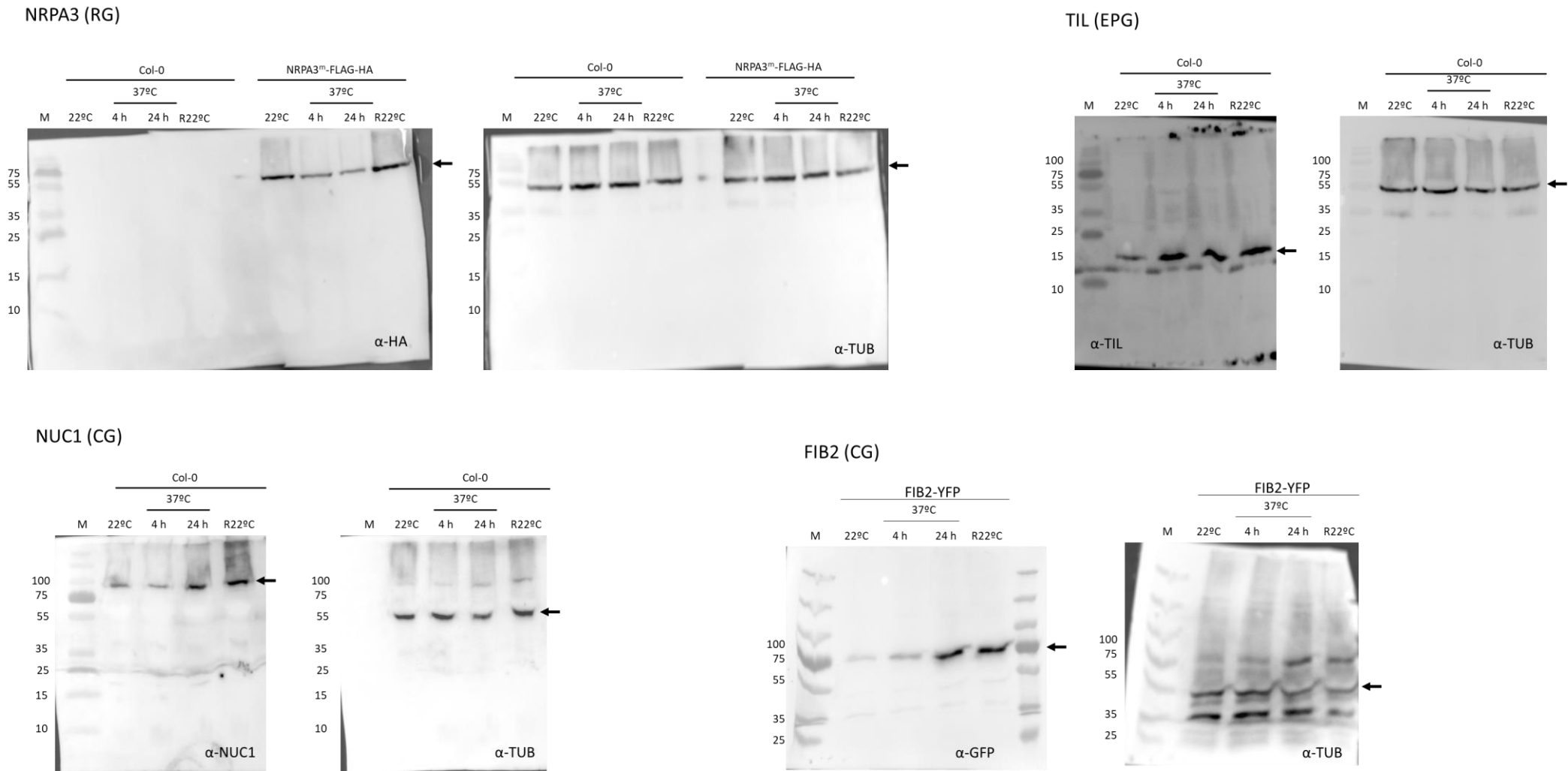

Supplement: Supplementary file 20 — Supplementary Information 20. [file 41598_2024_65558_MOESM20_ESM.pdf]
