# Supplementary material for: Proteomic profiling of Arabidopsis nuclei reveals distinct protein accumulation kinetics upon heat stress
Source: Sci Rep. 2024 Aug 14;14:18914. doi: 10.1038/s41598-024-65558-4 (PMC11324732; doi:10.1038/s41598-024-65558-4)

Figure S5A

Early-Like Group (ELG)

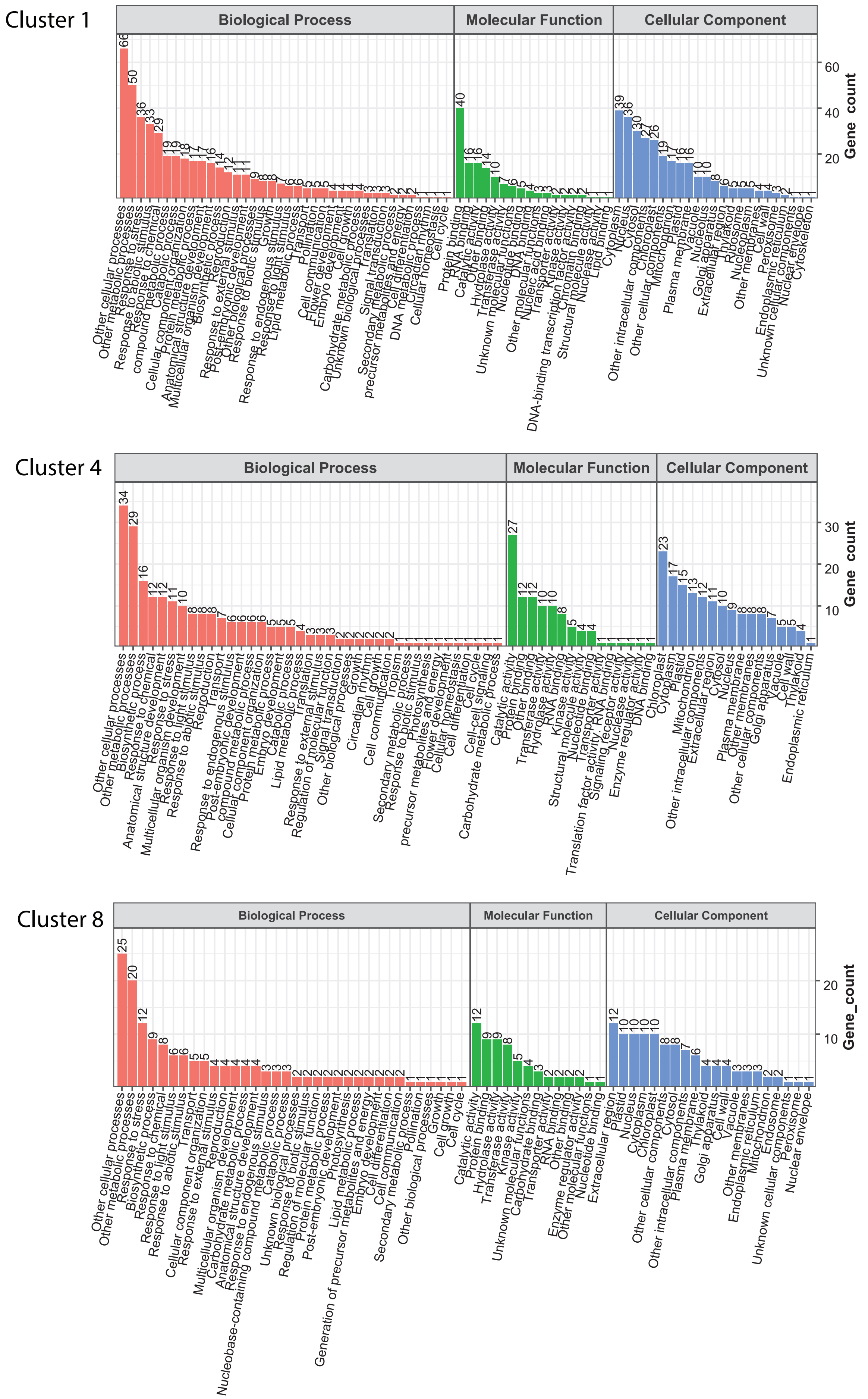

Supplement: Supplementary file 9 — Supplementary Information 9. [file 41598_2024_65558_MOESM9_ESM.pdf]
