# Supplementary material for: Proteomic profiling of Arabidopsis nuclei reveals distinct protein accumulation kinetics upon heat stress
Source: Sci Rep. 2024 Aug 14;14:18914. doi: 10.1038/s41598-024-65558-4 (PMC11324732; doi:10.1038/s41598-024-65558-4)
Supplement: Supplementary file 17 — Supplementary Information 17. [file 41598_2024_65558_MOESM17_ESM.pdf]

Supplemental Figures and Tables

Figure S1. NRPA3^{m}-FLAG-HA protein expression in *NRPA3$_{pro}$:NRPA3^{m}-FLAG-HA* (*nrpa3)* lines. **A)** Top, scheme of *NRPA3* (At1g60850) gene shows T-DNA insertion in SALKseq_088247 line and *NRPA3* genomic sequence cloned into a pCambia vector to obtain NRPA3^{m} fused to FlagFlag and HAHA sequences. Bottom, NRPA3-FLAG-HA protein sequence. The three cysteine residues mutated into serine and the 2X FlagHA sequence in the C-terminus sequence are shown **B)** Western blot analysis of Col-0 (lane 1) and four *nrpa3* plant lines transformed with the plasmid construct NRPA3^{m}-FLAG-HA (lanes 2-5).

Figure S2. Heat maps of 522 and 149 differentially accumulated proteins in the nuclear proteome. Heat map shows clustering (C1-C10) of the 522 **A)** or 149 **B)** differentially accumulated proteins a in the nucleus at 22 °C, 37 °C (4 h and 24 h) and R22 °C.

Figure S3A. Gene Ontology (GO) analysis. GO annotation with the Biological Process (red bars), Molecular Function (green bars) and Cellular Component (blue bars) of Early (EG), Late (EG) and Transient (TG) Groups.

Figure S3B. Gene Ontology (GO) analysis. GO annotation with the Biological Process (red bars), Molecular Function (green bars) and Cellular Component (blue bars) of Early Persistent (EPG), Late Persistent (LPG), and Recovery (RG) Groups.

Figure S4A. Heat map. Heat maps show the $\log_2$ FC for every protein from Clusters C1, C4 and C8 from Early-Like Group (ELG) in indicated temperature/time comparisons. *p* values for 37 °C 4 h vs 22 °C=0.0005); 37 °C 24 h vs 22 °C=0.001; R22 °C vs 22 °C=0.001), 37 °C 24 h vs 37 °C 4 h=0.0004, R22 °C vs 37 °C 4 h = 0.0007 and R22 °C vs 37 °C 24 h= 0.0005. FDR = 0.98-1.14%. Proteins whose nuclear abundance increases or decreases during exposure to 37 ºC are shown in yellow and blue respectively.

Figure S4B. Heat map. Heat maps show the $\log_2$ FC for every protein from Clusters C2, C3 and C7 from Late-Like Group in indicated temperature/time comparisons. *p* values for 37 °C 4 h vs 22 °C=0.0005); 37 °C 24 h vs 22 °C=0.001; R22 °C vs 22 °C=0.001), 37 °C 24 h vs 37 °C 4 h=0.0004, R22 °C vs 37 °C 4 h = 0.0007 and R22 °C vs 37 °C 24 h= 0.0005. FDR = 0.98-1.14%. Proteins whose nuclear abundance increases or decreases during exposure to 37ºC are shown in yellow and blue respectively.

Figure S4C. Heat map. Heat maps show the $\log_2$ FC for every protein from Cluster C6 from Transient-Like Group (TLG) in indicated temperature/time comparisons. *p* values for 37 °C 4 h vs 22 °C=0.0005); 37 °C 24 h vs 22 °C=0.001; R22 °C vs 22 °C=0.001), 37 °C 24 h vs 37 °C 4 h=0.0004, R22 °C vs 37 °C 4 h = 0.0007 and R22 °C vs 37 °C 24 h= 0.0005. FDR = 0.98-1.14%. Proteins whose nuclear abundance increases or decreases during exposure to 37 ºC are shown in yellow and blue respectively.

Figure S4D. Heat map. Heat maps show the log_2 FC for every protein from Cluster C5 from Continuous Group (CG) in indicated temperature/time comparisons. *p* values for 37 °C 4 h vs 22 °C=0.0005); 37 °C 24 h vs 22 °C=0.001; R22 °C vs 22 °C=0.001), 37 °C 24 h vs 37 °C 4 h=0.0004, R22 °C vs 37 °C 4 h = 0.0007 and R22 °C vs 37 °C 24 h= 0.0005. FDR = 0.98-1.14%. Proteins whose nuclear abundance increases or decreases during exposure to 37 ºC are shown in yellow and blue respectively.

Figure S5A. **Gene Ontology (GO) analysis of the four additional groups**. GO annotation with the Biological Process (red bars), Molecular Function (green bars) and Cellular Component (blue bars) of Early-Like Group (ELG) clusters 1, 4 and 8.

Figure S5B. **Gene Ontology (GO) analysis of the four additional groups**. GO annotation with the Biological Process (red bars), Molecular Function (green bars) and Cellular Component (blue bars) of Late-Like Group (LLG) clusters 2, 3 and 7.

Figure S5C. **Gene Ontology (GO) analysis of the four additional groups**. GO annotation with the Biological Process (red bars), Molecular Function (green bars) and Cellular Component (blue bars) of Transient-Like Group (TLG) cluster 6.

Figure S5D. **Gene Ontology (GO) analysis of the four additional groups**. GO annotation with the Biological Process (red bars), Molecular Function (green bars) and Cellular Component (blue bars) of Continuous Group (CG) cluster 5.

Figure S6. Nuclear proteome versus transcriptome under heat stress in Arabidopsis. Bar plots show the proportion of genes with positive correlation (0.5 to 1), negative correlation (-1 to -0.5) and non-correlation (-0.5 to 0 and 0 to 0.5) within the six difference comparisons (37 °C 4 h vs 22 °C; 37 °C 24 h vs 22 °C, R22 °C vs 22 °C, 37 °C 24 h vs 37 °C 4 h, 37 °C 4 h vs R22 °C and 37°C 24 h vs R22 °C).

Figure S7. Western blot using total protein extracts from Col-0, *$NRPA3_{pro}$:$NRPA3^{m}$-FLAG-HA* (in *nrpa3*), and *$35S_{pro}$:FIB2-YFP* (in Col-0) plants at 22 °C, 37 °C (4 h and 24 h) and R22 °C. Western blots were performed using α-HA, α-NUC1, α-GFP, and α-TIL1 to detect $NRPA3^{m}$-FLAG-HA (RG), NUC1, FIB2-YFP (CG) and TIL1 (EPG) proteins, respectively. α-TUBULIN (TUB) was used to verify protein loading and quantification (using ImageJ software) of the $NRPA3^{m}$-FLAG-HA, NUC1, FIB2-YFP, TIL1 and TUB band intensities. Ratio of band intensity for each protein versus TUB at 22 °C, 37 °C (4 h and 24 h) and R22 °C are show (Table S9). Expected sizes for $NRPA3^{m}$-FLAG-HA ~50 kDa, NUC1: ~60 kDa; FIB2-YFP: ~65 kDa; TIL1: ~21 kDa and TUB: ~50 kDa are indicated.

Figure S8. Gene Ontology (GO) analysis. Biological Process of the 522 differentially accumulated proteins (Table S4. The most relevant Biological Process are zoomed in a, b and c. Generated with http://bioinfo.cau.edu.cn/agriGO/

Figure S9. Intrinsically Disorder Regions (IDR) *in silico* of NRPA3, NUC1, FIB2, NOP56 and GAR1. IDRs have been described to drive the LLPS behavior of the nucleolus. FIB2 from the C/D Box complex displays a well-documented disordered region in its N-terminal domain. The major nucleolar factor NUC1 have tendency to disorder in its N-terminal, but also the C-terminal (GAR) domain. Similarly NOP56 found in the C/D box has region disorder in N- and -C terminal domains. GAR1 protein found in the H/ACA complexes has region disorder in C-terminal domains. In contrast, NRPA3 has not disordered region. The intrinsically disordered tendency was calculated by IUPred algorithm (www.iupred2a.elte.hu, accessed on August, 4 2023), a tendency to disorder is predicted when values per residue exceed the 0.5 IUPred scores.

Table S1: List of proteins detected by LC-MSMS à 22 °C, 37 °C 4 h, 37 °C 24 h and after 24 h of recovery at 22 °C (R22 °C).

Table S2: LC_MSMS raw data.

Table S3: Abundance of peptides from 2629 Arabidopsis accessions à 22 °C (replicas 1-3), 37 °C 4 h (replicas 1-3), 37 °C 24 h (replicas 1-3) and after 24 h of recovery at 22 °C (R22 °C) (replicas 1-3).

Table S4: List of 522 differentially accumulated proteins. Highlighted in blue are proteins with GO Cellular component associated with the nucleus, nucleolus, nucleoplasm, or nuclear envelope. The identification of TFs was carried out with AGRIS (https://agris-knowledgebase.org/) and the NLS prediction was performed with the LOCALIZER online tool (https://localizer.csiro.au/).

Table S5: Statistical criteria and list of proteins from the Early Group (EG), Late Group (LG), Transient Group (TG), Early Persistent Group (EPG), Late Persistent Group (LPG) and Recovery Group (RG).

Table S6: List of proteins from Early-Like Group (ELG_C1, ELG_C4 and ELG_C8); Late-Like Group (LLG_C2, LLG_C3 and LLG_C7); Transient-Like Group (TLG_C6); and Continuous Group (CG_C5).

Table S7: RNAseq analysis of Arabidopsis plants untreated (22 °C), heat treated (37 °C, 2 h, 5 h, 24 h) and recovered at 22 °C after 24 h at 37 °C (R22_5h and R22_24h).

Table S8: Pearson correlation coefficients for each protein gene and used in Figures 5 and S8.

Table S9: Quantification of NRPA3^{m}-FLAG-HA, NUC1, FIB2-YFP, TIL1 and TUB (using ImageJ software) band intensities detected by western blot (Figure S7) and TUB peptide detected by LC-MSMS (Table S3, tab Feature Meta Data)
