# Supplementary material for: Proteomic profiling of Arabidopsis nuclei reveals distinct protein accumulation kinetics upon heat stress
Source: Sci Rep. 2024 Aug 14;14:18914. doi: 10.1038/s41598-024-65558-4 (PMC11324732; doi:10.1038/s41598-024-65558-4)
Supplement: Supplementary file 18 — Supplementary Information 18. [file 41598_2024_65558_MOESM18_ESM.docx]

# Supplementary Material & Method

## Proteomic analysis

### Electrophoresis and in gel trypsin digestion

Purified nuclei were diluted four times in water to reach 1X Laemmli buffer. 32 µl were loaded on an in-house poured 4% acrylamide stacking gel. Gel was stained with Coomassie Blue and the stacking bands were manually excised. Proteins were then reduced, alkylated and digested overnight at room temperature with modified trypsin at a 1:100 enzyme:protein ratio (Promega, Madison, USA). Peptides were extracted during 1 hour with 90 μL of 80% acetonitrile, 0.1% formic acid. Peptide mixtures were then dried and resuspended in water acidified with 0.1% formic acid.

### Liquid Chromatography-Tandem Mass Spectrometry (LC-MS/MS) Analyses

LC-MS/MS analyses of peptide extracts were performed on a NanoAcquity LC-system (Waters, Milford, MA, USA) coupled to a Q-Exactive plus Orbitrap (Thermo Fisher Scientific, Waltham, MA, USA) mass spectrometer equipped with a nanoelectrospray ion source. Mobile phase A (99.9% water and 0.1% FA) and mobile phase B (99.9% acetonitrile and 0.1%FA) were delivered at 400 nl/min. Samples were loaded into a Symmetry C18 precolumn (0.18 x 20 mm, 5 μm particle size, Waters) over 3 minutes in 1% buffer B at a flow rate of 5 μL/min. This step was followed by reverse-phase separation at a flow rate of 400 nl/min using an ACQUITY UPLC® BEH130 C18 separation column (250 mm x 75 μm id, 1.7 μm particle size, Waters). 1 µl of peptide mixtures were eluted using a gradient from 1% to 35% B in 79 minutes, from 35% B to 90% B in 1 minute, maintained at 90% B for 5 minutes and the column was reconditioned at 1% B for 20 minutes.

The Q-Exactive Plus Orbitrap instrument was operated in data dependent acquisition mode by automatically switching between full MS and consecutive MS/MS acquisitions. Survey full scan MS spectra (mass range 300-1800) were acquired with a resolution of 70,000 at 200 m/z with an automatic gain control (AGC) fixed at 3 x 10^6^ ions and a maximum injection time set at 50 ms. The ten most intense peptide ions in each survey scan with a charge state >= 2 were selected for MS/MS fragmentation. MS/MS scans were performed at 17,500 resolution at 200 m/z with a fixed first mass at 100 m/z, AGC was fixed at 1 x 10^5^ and the maximum injection time was set to 100 ms. Peptides were fragmented by higher-energy collisional dissociation (HCD) with a normalised collision energy set to 27. Peaks selected for fragmentation were automatically put on a dynamic exclusion list for 60 s and peptide match selection was turned on. MS data were saved in .raw file format (Thermo Fisher Scientific) using XCalibur.

### LC-MS/MS data interpretation and validation

Raw files were converted to .mgf peaklists using MsConvert (using MSAngel) and were submitted to Mascot database searches (version 2.6.2, MatrixScience, London, UK) against an *Arabidopsis thaliana* protein sequences database downloaded from *The Arabidopsis Information Resource TAIR* site (TAIR10 version gene model 2011/01/03), common contaminants and decoy sequences were added. The concatenated database contains 2 x 27 534 protein entries. Spectra were searched with a mass tolerance of 5 ppm in MS mode and 0.07 Da in MS/MS mode. One trypsin missed cleavages was tolerated. Carbamidomethylation of cysteine residues was set as a fixed modification. Oxidation of methionine residues and acetylation of protein n-termini were set as variable modifications. Identification results were imported into Proline software (http://proline.profiproteomics.fr/) for validation. Peptide Spectrum Matches (PSM) with pretty rank equal to one were retained. False Discovery Rate was then optimized to be below 1% at PSM level using Mascot Adjusted E-value and below 1% at Protein Level using Mascot Mudpit score. Only protein sets with at least a specific peptide were retained ^1^.

### Label Free Quantification and Statistical Analysis

*Label Free Quantification*

Peptides Abundances were extracted thanks to Proline software version 2.0 (<http://proline.profiproteomics.fr/>) using a m/z tolerance of 5 ppm. Alignment of the LC-MS runs was performed using Loess smoothing. No Cross Assignment was performed. The best 2+ or 3+ peptide ion was used to assign an abundance to a peptide. Peptides abundances were normalized using the median across the 12 runs. Protein Abundance was then computed by summing the abundance of all the peptides except the one with a doubtful peak assignation (different peptides identification sharing the same speak).

*Statistical Analysis*

Protein Abundances were loaded into Prostar software version 1.18.6 (<http://www.prostar-proteomics.org/>) and associated to their conditions (22 °C, 37 °C 4h, 37 °C 24h, R22 °C). Proteins with at least 3 values in at least one condition (3/3 replicates) were kept for further statistical analysis. Contaminants were removed. Residual Missing Values were imputed in a conservative way (quantile 2.5%). Pairwised Limma t-tests were performed.

P-values calibration was corrected using adapted Benjamini-Hochsberg method, and FDR was set to ~1%. More precisely, the comparison of 22 °C vs 37 °C 24h lead to 1.00% FDR using p-values below 0.00132, the comparison of 22 °C vs 37 °C 4h lead to 1.03% FDR using p-values below 0.00501, the comparison of 22 °C vs R22 °C lead to 1.01 % FDR using p-values below 0.00955, the comparison of 37 °C 4h vs 37°C 24h lead to 1.14 % FDR using p-values below 0.00389, the comparison of 37 °C 4h vs R22 °C lead to 1.02 % FDR using p-values below 0.00692, and finally the comparison of 37 °C 24h vs R22 °C lead to 0.98 % FDR using p-values below 0.00479.

1 Bouyssie, D. *et al.* Proline: an efficient and user-friendly software suite for large-scale proteomics. *Bioinformatics (Oxford, England)* **36**, 3148-3155 (2020). <https://doi.org:10.1093/bioinformatics/btaa118>

2 Durut, N. *et al.* A duplicated NUCLEOLIN gene with antagonistic activity is required for chromatin organization of silent 45S rDNA in Arabidopsis. *The Plant Cell* **26**, 1330-1344 (2014). <https://doi.org:10.1105/tpc.114.123893>

3 Pontvianne, F. *et al.* Nucleolin is required for DNA methylation state and the expression of rRNA gene variants in Arabidopsis thaliana. *PLoS Genetics* **6**, e1001225 (2010). <https://doi.org:10.1371/journal.pgen.1001225>

4 Chi, W. T., Fung, R. W., Liu, H. C., Hsu, C. C. & Charng, Y. Y. Temperature-induced lipocalin is required for basal and acquired thermotolerance in Arabidopsis. *Plant Cell Environ* **32**, 917-927 (2009). <https://doi.org:10.1111/j.1365-3040.2009.01972.x>

5 Montacie, C. *et al.* Nucleolar Proteome Analysis and Proteasomal Activity Assays Reveal a Link between Nucleolus and 26S Proteasome in A. thaliana. *Front Plant Sci* **8**, 1815 (2017). <https://doi.org:10.3389/fpls.2017.01815>

## Western blotting

Total proteins were extracted from 100 mg of 15-day-old non-treated (22 °C), heat-treated (37 °C 4 h and 37 °C 24 h) and recovered (R22 °C) seedlings. Then, plant material was ground to a fine powder in a mortar with liquid nitrogen. The ground samples were resuspended in 300 µl of 2X Laemmli buffer and incubated at 65 °C for 10 min. After centrifugation at 14,000 × g for 15 min at 4 °C, the supernatant was retained as the total protein extract. Fifteen microlitres of total protein extract separated by SDS‒PAGE, followed by Western blot analyses as previously described ^2^. Membranes were hybridized either with a 1:5,000 dilution of α-NUC1 ^3^ of α-TIL1 ^4^ to detect NUC1 and TIL1 respectively in Col-0, a 1:5,000 dilution of α-GFP (Rockland) to detect FIB2-YFP in Col-0-transformed *35S_pro_:FIB2-YFP* ^5^, a 1:10,000 dilution of α-HA-HRP to detect NRPA3^m^-FLAG-HA in *nrpa3*-transformed *NRPA3_pro_:NRPA3^m^-FLAG-HA*, or with a 1:100,000 dilution of α-Tubulin (Agrisera). The Western blot bands were analysed with ImageJ.
